# KROX20 marks sebaceous gland stem cells and regulates their differentiation to maintain skin oil balance

**DOI:** 10.1172/JCI206364

**Published:** 2026-09-22

**Authors:** Yumeng Zhang, Pernelle Pulh, Michelle F. Pan, Yi He, Juanzhu Yan, Annie Li, Renée M. McKay, Lu Q. Le

**Affiliations:** 1 Department of Dermatology, University of Virginia, Charlottesville, Virginia, USA.; 2 Department of Cell Biology, University of Texas Southwestern Medical Center, Dallas, Texas, USA

**Keywords:** Cell biology, Development, Adult stem cells, Skin, Stem Cells

## Abstract

Oil-producing sebaceous glands (SGs), attached to the upper/middle portion of hair follicles, are indispensable for maintaining skin hydration, and their dysfunction leads to dry skin. However, the molecular mechanisms underlying regulation of SG stem cells remain largely unknown. We identified transcription factor KROX20 as a marker of SG stem cells that sustains their stemness throughout SG morphogenesis and homeostasis. We developed an inducible mouse model in which ablation of KROX20-positive cells causes SG loss and rapidly and robustly induces dry, flaky, alopecia symptoms following induction. This model, termed xeroflacia (xero = xerosis, fla = flaky, cia = alopecia), provides a tool for studying the biology of dry skin. Furthermore, we found that KROX20 directly regulates *Notch1* transcription to orchestrate the balance between SG stem cell self-renewal and differentiation. Small molecule drug modulation of *Notch1* signaling to activate or inhibit the pathway enabled us to regulate SG differentiation to maintain skin oil levels, providing a proof of principle that other signaling pathways downstream of *Krox20* could be therapeutic targets for SG-related diseases.

## Abstract 2

We identified the transcription factor KROX20 as a sebaceous gland stem cell marker that is required for their self-renewal and differentiation.

## Graphical abstract

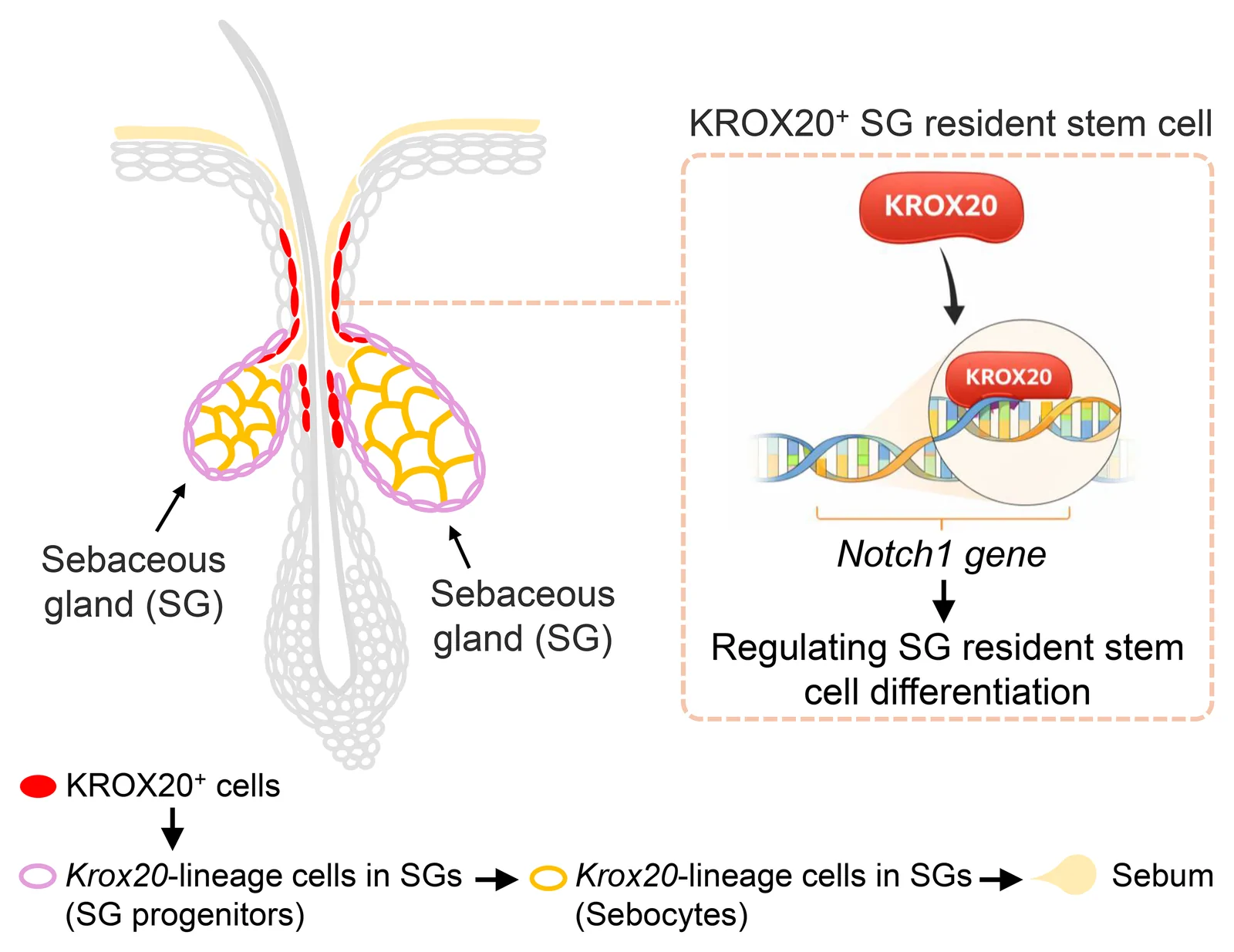

## Introduction

Skin serves as the primary barrier enabling humans to adapt to and survive in an ever-changing external environment, and hair follicle–associated sebaceous glands (SGs) are indispensable for proper skin protection (1). SGs are holocrine glands containing differentiated sebocytes that accumulate lipids, disintegrate, and release their contents (sebum) onto the skin surface to form the lipid film (2). In addition to lipid production, accumulating evidence indicates that SGs are essential for antimicrobial defense through the expression of sebum-associated antimicrobial peptides (AMPs) (3, 4). Among AMPs, small proline-rich proteins (SPRR1 and SPRR2) are expressed in sebocytes, and these AMPs exert bactericidal activity by directly binding to and disrupting bacterial membranes, thereby protecting the host from systemic infection (4). Therefore, during evolutionary processes, SGs may provide a mechanism for organisms to adapt more rapidly to environmental changes and challenges. Beyond physiological roles, SGs also contribute to various pathophysiological processes: reduced sebum production has been linked to dry skin and atopic dermatitis (5), while excessive sebum secretion is associated with several skin disorders, including acne vulgaris (6).

Mouse SG morphogenesis initiates around embryonic day 17.5, marked by the expression of SG-specific peroxisome proliferator–activated receptor γ (PPARγ) in developing hair buds (7), and nascent SGs continue to develop to reach mature size around postnatal day 7 (8). Once fully formed, SGs are constantly renewed during homeostasis, with differentiated/ruptured sebocytes being replenished and replaced by proliferative progenitor cells residing in the SG basal layer at the periphery of each SG lobe (8). Therefore, the constant turnover of sebocytes underscores the existence of a resident stem cell pool, and recent research has provided evidence that each individual lobe of the typical 2-lobed SG structure is maintained by its own resident stem cell pool (9). Emerging studies have shown that SG resident stem cells reside in the junction area between the upper/middle hair follicle and the SG lobe (10, 11), referred to as the “SG root” in this report. Although a few SG resident stem cell markers, such as *Lrig1* (12–14) and *Gata6* (15, 16), have been reported, the molecular mechanisms regulating SG resident stem cells during development and homeostasis remain unclear. We previously reported that the transcription factor KROX20 is expressed in cells residing within the upper/middle hair follicle in mice and that *Krox20*-lineage cells contribute to the hair follicle bulge (17) and interfollicular epidermis (IFE) (18, 19). Recent human spatial transcriptomics studies enabled examination of *KROX20* expression in human skin, revealing a distribution pattern comparable to that observed in mice (20, 21). Furthermore, we showed that KROX20 is expressed in the ductules of Meibomian glands (MGs), specialized SGs within the tarsal plates of eyelids: *Krox20*-lineage cells give rise to the entire MG (22), suggesting that KROX20 could be involved in regulating self-renewal maintenance and differentiation of resident stem cells of holocrine glands. In this study, we found that loss of *Krox20* results in SG depletion. Furthermore, ablation of KROX20-positive cells rapidly and robustly induces dry, flaky, alopecic skin with 100% penetrance in these mice; we call this phenotype “xeroflacia” (xero = xerosis, fla = flaky, cia = alopecia).

As an evolutionarily conserved pathway, *Notch* signaling has been reported to be essential for skin development and homeostasis (9, 23–25). Although the *Jag2*/*Notch1* signaling axis is a key regulator of SG resident stem cell differentiation (26), the molecular mechanism by which *Notch1* signaling is regulated in SG stem cells is not fully understood. Here, we found that KROX20 can modulate *Notch1* signaling in SG resident stem cells by transcriptionally regulating the *Notch1* gene. Overall, we identified a SG stem cell marker, KROX20, and found that it is indispensable for maintaining the self-renewal and differentiation of SG stem cells.

## Results

### Deletion or overexpression of Krox20 impairs SG morphogenesis.

SGs are holocrine glands in the skin, commonly presenting as 2 symmetric lobular structures (2-SG) linked to the infundibulum of the hair follicle via the SG root. To determine the role of *Krox20* in SGs, the *Krox20* gene was conditionally deleted: *Krox20^fl/fl^ K14-Cre* (*Krox20-cKO*) mice (Figure 1A). To avoid the sectional bias that can occur when evaluating stereoscopic tissue, whole-mount SG visualization was performed on tail and dorsal skin to accurately present SG architecture using the lipophilic fluorescent dye Nile Red (9, 27). Nile Red staining of tail skin revealed severely atrophied or nearly complete loss of SG lobes in 4-month-old (4M) *Krox20-cKO* mice (Figure 1B and Supplemental Videos 1 and 2; supplemental material available online with this article; https://doi.org/10.1172/JCI206364DS1). Quantitative analyses of the whole-mount images confirmed that the percentage of hair follicles with SGs was significantly lower in *Krox20-cKO* mice compared with littermates (Figure 1C). We next evaluated the number of hair follicles bearing 2 SG lobes (2-SG), 1 SG lobe (1-SG), and no SG lobes (0-SG): compared with littermates, *Krox20-cKO* mice showed a significantly higher proportion of hair follicles with 0-SG and 1-SG, accompanied by a corresponding reduction in follicles with 2-SG (Figure 1D), and a reduced average number of SG lobes per follicle (Figure 1E). Furthermore, the average length and average area of SG lobes were significantly reduced in *Krox20-cKO* mice compared with littermates (Figure 1, F and G). Similar SG morphological changes were also evident in dorsal skin of *Krox20-cKO* mice (Supplemental Figure 1, A–F). To evaluate functional changes of the SGs, we extracted the oleaginous substances from hair and analyzed their lipid composition using thin-layer chromatography (TLC). In *Krox20-cKO* mice, lipid output was diminished across multiple classes, including cholesterol ester, wax ester (WE), free fatty acid, and free cholesterol, with a particularly notable reduction in the sebum-specific WE, suggesting a diminished lipid film over the outermost epidermal surface (Supplemental Figure 1G). As a result, the reduced lipid film in *Krox20-cKO* mice led to elevated TEWL and TEHL (Figure 1H). These data indicated that deletion of *Krox20* caused a pronounced loss of SGs and that *Krox20* may play a key role in SG morphogenesis.

In addition to the 4M stage, we asked whether *Krox20* deficiency results in SG loss at earlier stages. SG morphology was assessed at P22, P27, P32, and P40 (Supplemental Figure 1H). In littermate mice, the proportion of hair follicles bearing SGs and the average number of SG lobes per hair follicle remained constant from P22 to P40, while in *Krox20-cKO* mice, these declined gradually (Supplemental Figure 1, I and J). Interestingly, SG lobes in littermates and *Krox20-cKO* mice displayed a K-shaped difference in both average length and area: SGs continued to grow from P22 to P40 in littermate mice, whereas *Krox20-cKO* mice gradually lost their SGs (Supplemental Figure 1, K and L).

We next wanted to determine the effects of overexpressing *Krox20* in SGs. To this end, a noninducible *Cre* system was employed to drive *Krox20* overexpression specifically in *Krox20*-expressing cells in *Krox20-Cre R26-Krox20 R26-tdTomato* (*Krox20-OE*) mice (Figure 1I). Compared with 2M littermates, analysis of tail skin SGs from 2M *Krox20-OE* mice revealed an atrophic SG morphology (Figure 1J and Supplemental Videos 3 and 4). Although there was no significant difference between 2M littermates and 2M *Krox20-OE* mice regarding the distribution of hair follicles bearing 0-SG, 1-SG, 2-SG, 3-SG, and multi-SG and average number of SG lobes per follicle (Supplemental Figure 2, A and B), *Krox20-OE* mice displayed significantly decreased SG lobe length and area (Supplemental Figure 2, C and D), decreased WE production (Supplemental Figure 2E), and increased TEWL and TEHL (Supplemental Figure 2F) relative to littermates, suggesting reduced sebum production. The SG morphological changes seen in tail skin were also observed in dorsal skin (Supplemental Figure 2, G–L). These findings suggest that *Krox20* likely maintains the stemness of SG stem cells: its overexpression blocks SG stem cells from becoming differentiated sebocytes, ultimately causing SG atrophy. Strikingly, 4 months later, 6M *Krox20-OE* mice exhibited multilobular SG morphology compared with 6M littermates (Figure 1K and Supplemental Videos 5 and 6), along with significantly higher frequency of follicles bearing 3-SG or multi-SG (Supplemental Figure 2M) and a greater average number of smaller SG lobes per follicle (Supplemental Figure 2, N–P). These data suggest that *Krox20*, acting as a potential stemness maintainer in SG stem cells, potentiates ectopic multilobular SG formation in the infundibulum when overexpressed. Functionally, no differences were observed between 6M littermates and 6M *Krox20-OE* mice in WE production (Supplemental Figure 2Q), TEWL, and TEHL (Supplemental Figure 2R), indicating that *Krox20-OE* mice may counterbalance SG atrophy and diminished sebum production by expanding SG number (Figure 1L), highlighting the skin’s intrinsic capacity to maintain tissue homeostasis.

Collectively, these data indicate that KROX20 maintains the stemness of SG resident stem cells. Loss of *Krox20* depletes the SG stem cell pool, resulting in SG lobe loss. In contrast, *Krox20* overexpression initially hyperactivates SG stem cells and prevents their differentiation into sebocytes, leading to SG atrophy, and subsequently promotes ectopic multilobular SG formation as a compensatory response to SG atrophy. These observations prompted us to investigate the mechanisms by which KROX20 regulates SG resident stem cells.

### KROX20 is a marker of SG resident stem cells and preserves their stemness.

To systematically evaluate *Krox20*-expressing cells in the pilosebaceous unit, we reanalyzed a publicly available SG-specific scRNA-seq dataset derived from PPARγ-lineage cells (7). Within the SG-specific PPARγ-lineage cells, *Krox20* mRNA was enriched at the junction between upper hair follicle cells and SG basal cells, which reside in the peripheral layer of SGs. This junctional region has been defined as the SG root and a niche for SG resident stem cells (10, 11) (Figure 2A), suggesting that *Krox20* may serve as a marker of SG resident stem cells. Next, we assessed expression of KROX20 and SG stem/progenitor marker LRIG1 in SGs from littermates (4M) and *Krox20-cKO* (4M) mice (Figure 2B). Compared with littermates, *Krox20-cKO* mice exhibited a complete loss of KROX20^+^ cells in SGs, with a concomitant ~50% decrease in LRIG1^+^ cells relative to littermates (Figure 2C). The diagram in Figure 2D illustrates the distribution changes of KROX20^+^LRIG1^–^, KROX20^+^LRIG1^+^, and KROX20^–^LRIG1^+^ cells in *Krox20-cKO* mice, indicating that the loss of KROX20^+^ cells in the SG root was a key driver of SG lobular loss/atrophy (Figure 2D), accompanied by a significant reduction in Ki67^+^ cells and PPARγ^+^ cells in SGs (Figure 2, E and F).

Conversely, *Krox20-OE* mice showed a significant increase in KROX20^+^ cells in SGs, accompanied by a 3-fold increase in the number of KROX20^+^LRIG1^+^ cells compared with littermates (Figure 2, G and H, and Supplemental Figure 3, A–C), indicating an accumulation of SG resident stem cells and suppression of downward differentiation (Figure 2I). Like *Krox20-cKO* mice, a significant decrease in Ki67^+^ proliferative cells and PPARγ^+^ SG basal cells was also observed in *Krox20-OE* mice (Figure 2, J and K, and Supplemental Figure 3D); however, the underlying mechanisms differed: in *Krox20-cKO* mice, loss of KROX20^+^ cells depleted the SG resident stem cell pool for downstream progenitors, resulting in SG lobular loss or atrophy, whereas in *Krox20-OE* mice, ectopic activation of KROX20 maintained SG resident stem cell stemness, inhibiting downstream differentiation and leading to SG atrophy. Taken together, KROX20 and LRIG1 expression in SGs under *Krox20*-knockout and -overexpression scenarios suggested that KROX20 serves as a marker of SG resident stem cells and preserves their stemness.

### KROX20 marks SG stem cells, and Krox20-lineage cells contribute to SG morphogenesis.

To confirm the essential role of KROX20 in SG stem cells, *Krox20*-lineage tracing was performed. Lineage tracing would provide robust evidence of the dynamic contribution of KROX20^+^ cells and their progeny to SGs, delineating a clear lineage pathway from SG stem cells to their differentiated descendants, sebocytes. First, we used a constitutive Cre recombinase system to determine whether *Krox20*-lineage cells globally contribute to the developing SG (dSG) and mature SG (mSG): *Rosa26-tdTomato* reporter mice were crossed with mice expressing Cre recombinase under the control of the *Krox20* expression (*Krox20-Cre R26-tdTomato*) (28, 29). We examined dSG at P3 and P5 and mSG at P7. Due to the immature state of SGs before P7, the typical SG lobular morphology was not yet apparent. Therefore, we assessed SGs using double staining for PPARγ and tdTomato, since PPARγ^+^ cells are specific to SGs within the pilosebaceous unit. In line with a previous study (8), dSG, characterized by lobe-like structures containing PPARγ^+^ cells, were situated near the IFE at P3 (Figure 3A). The distance between dSG and epidermis progressively increased from P3 to P7 due to infundibulum development. KROX20^+^ cells were consistently localized to the root region of both dSG and mSG, and SGs at both stages were tdTomato^+^, suggesting that KROX20 expression in the SG developing bud was initiated prior to P3 and that *Krox20*-lineage cells globally contributed to SG development (Figure 3A).

Next, we performed lineage tracing with an inducible Cre driver, considered the gold standard for lineage tracing analysis, to observe KROX20^+^ cells and the spatiotemporal dynamics of their progeny at the SG morphogenesis stage. *Rosa26-tdTomato* reporter mice were crossed with *Krox20-CreERT2* mice, in which the expression of CreERT2 recombinase is driven by the *Krox20* expression, to generate *Krox20-CreERT2 R26-tdTomato* mice (17). These mice were induced with 4-hydroxytamoxifen (4-HT) at P3 and then euthanized at P3 + 8 hours, P3 + 24 hours, P5, and P7 (Figure 3B). Double staining for KROX20 and tdTomato revealed that KROX20^+^ cells were consistently localized in the root region of both dSGs and mSGs, similar to observations in the noninducible system. No *Krox20*-lineage cells were detected at P3 (Figure 3C). However, 24 hours postinduction (P3 + 24h), tdTomato^+^ cells appeared in the SG basal layer, where SG progenitors reside, and subsequently contributed to the entire dSG by P5 and mSG by P7 (Figure 3, C and D). These findings suggested that SG morphogenesis depends on KROX20^+^ cells in the SG root (Supplemental Figure 4A), whose lineage gave rise sequentially to SG LRIG1^+^ progenitors (Supplemental Figure 4B) and FASN^+^ sebocytes (Supplemental Figure 4C), accompanied by tdTomato^+^ cells distributed throughout the entire SGs (Supplemental Figure 4D). To rule out potential leakiness of the inducible CreERT2 system, *Krox20-CreERT2; R26-tdTomato* mice without 4-HT induction were analyzed at the same time points (Supplemental Figure 4E). The absence of tdTomato signal at all time points confirmed tight 4-HT–dependent control of *Krox20-CreERT2* in SGs (Supplemental Figure 4F).

### KROX20^+^ cell ablation depletes SGs, leading to dry, flaky skin.

We next wanted to determine the role of KROX20^+^ cells in SG morphogenesis. To conditionally ablate KROX20^+^ cells, we crossed *Krox20-flox-eGFP-flox-DTA* knockin allele mice (in which the e*GFP* gene is flanked by 2 *LoxP* sites preventing transcription of the gene encoding diphtheria toxin A; DTA) with *K14-CreERT2* mice to generate *Krox20-GFP-DTA K14-CreERT2* (KROX20^+^ cell ablation) mice (30, 31). In this model, inducible CreERT2-mediated excision of the *eGFP* cassette permits *Krox20*-driven DTA expression following 4-HT induction, leading to selective ablation of KROX20^+^ cells (Figure 4A). *Krox20-GFP-DTA K14-CreERT2* mice were induced with 4-HT at P2, and skin phenotypes were continuously monitored thereafter (Figure 4A and Supplemental Figure 5A). Strikingly, ablation of KROX20^+^ cells in *K14*-expressing pilosebaceous units rapidly induced dry, flaky, alopecic skin, with symptoms appearing as early as 7 days postinduction (Supplemental Figure 5B). The phenotype was accompanied by elevated TEWL and TEHL relative to littermates, suggesting reduced sebum production (Supplemental Figure 5C). Fourteen days postinduction (P15), *Krox20-GFP-DTA K14-CreERT2* mice developed severe, widespread, dry, flaky, alopecia symptoms, affecting the dorsal, ventral, ear, and tail skin (Figure 4B). Whole-mount visualization of dorsal and tail SGs revealed that ablation of KROX20^+^ cells caused a profound loss of SGs (Figure 4, C–H), indicating that loss of KROX20^+^ SG stem cells caused the dry skin phenotype. We also histologically examined the P15 dorsal and tail skin from littermates and *Krox20-GFP-DTA K14-CreERT2* mice: KROX20^+^ cell ablation mice exhibited significantly increased epidermal thickness and reduced dermal white adipose tissue (dWAT) thickness in both dorsal and tail skin (Supplemental Figure 5, D–I), and epidermal thickening coincided with an elevated number of Ki67^+^ proliferative cells in the epidermis basal layer where epidermis stem cells reside (Supplemental Figure 5J). Compared with littermates, KROX20^+^ cell ablation dermis exhibited loss of fibronectin and collagen I (Supplemental Figure 5K). Based on this phenotype, we named this inducible KROX20^+^ cell ablation mouse model xeroflacia (xero = xerosis, fla = flaky, cia = alopecia).

Although ablation of KROX20^+^ SG stem cells rapidly triggered dry skin conditions, the underlying biological mechanisms remained elusive. We hypothesized that immune responses secondary to dry skin play a critical role in the development of xeroflacia symptoms. Compared with littermates, *Krox20-GFP-DTA K14-CreERT2* mice exhibited substantial infiltration of CD3^+^ T cells and CD8^+^ cytotoxic T cells into the epidermis and dermis, accompanied by a significant reduction in CD4^+^ helper T cells in these compartments (32) (Supplemental Figure 6A). F4/80^+^ phagocytic macrophages were also recruited to the epidermis and dermis (Supplemental Figure 6B). While iNOS^+^ M1 macrophages did not exhibit notable infiltration in either normal or xeroflacia skin, the presence of CD206^+^ M2 macrophages was significantly increased in the dermis of xeroflacia skin compared with controls (Supplemental Figure 6B). These findings support the participation of M2 macrophages but not M1 macrophages in phagocytosis and dWAT lipolysis, consistent with a previous report (33).

### KROX20 marks SG stem cells, and Krox20-lineage cells contribute to SG homeostasis.

Given the critical role of *Krox20*-lineage cells in SG morphogenesis, we next asked whether KROX20 remains a marker of SG resident stem cells in adult mice and whether *Krox20*-lineage cells contribute to SG homeostasis over the course of the mouse life span. To determine this, *Krox20-CreERT2; R26-tdTomato* mice were induced with tamoxifen at P21, then euthanized at P22, P29, and P40 to capture lineage dynamics within 24 hours (P22) and across a full SG turnover period of 7–14 days (P29 and P40) (2) (Figure 5A). Immunostaining revealed that KROX20 expression was restricted to the SG stem cell niche at the SG root, while *Krox20*-lineage cells appeared within 24 hours (P22), progressively contributing to SG progenitors in the basal layer by P29, and ultimately populating the entire SG by P40 (Figure 5B and Supplemental Figure 7A), thus demonstrating that *Krox20* marks SG resident stem cells in adult mice and that *Krox20*-lineage cells contribute to SG homeostasis (Figure 5C). Lack of CreERT2 leakiness was also confirmed in the adult *Krox20-CreERT2 R26-tdTomato* mice (Supplemental Figure 7, B and C). Furthermore, we also tested whether *Krox20*-lineage cells contribute to SG homeostasis long-term. *Krox20-CreERT2 R26-tdTomato* mice were induced with 4 mg tamoxifen for 4 days from P31 to P34 and subsequently sacrificed at adult (13M) and aged (18M) stages (Figure 5D). *Krox20*-lineage cells occupied the entire SG at both stages, with KROX20 expression confined to the SG stem cell niche (Figure 5E). Assessment of CreERT2 leakiness further validated the stringent control of the *Krox20-CreERT2 R26-tdTomato* system throughout these long-term experiments (Supplemental Figure 7, D and E).

Ablation of KROX20^+^ SG stem cells is essential to prove that KROX20^+^ cells give rise to the SG during SG homeostasis. To selectively ablate KROX20^+^ SG stem cells, we crossed mice carrying the *Krox20-flox-eGFP-flox-DTA* knockin allele with *Krox20-CreERT2* mice to generate *Krox20-GFP-DTA Krox20-CreERT2* mice (KROX20^+^ cell ablation). We induced mice with tamoxifen for 4 days from P21 through P24 and analyzed the mice at P26 (Figure 5F). Whole-mount imaging of dorsal skin showed atrophied SGs in *Krox20-GFP-DTA Krox20-CreERT2* mice compared with littermates; many hair follicles had no SG lobes (Figure 5G), and existing SG lobes were significantly reduced in length and area (Figure 5, H and I). Similarly, tail skin SGs from *Krox20-GFP-DTA Krox20-CreERT2* mice were atrophied (Figure 5, J–L).

In summary, these analyses of KROX20 expression, *Krox20*-lineage tracing, and KROX20^+^ cell ablation during SG development and homeostasis support that KROX20 marks SG resident stem cells and is indispensable for SG morphogenesis and homeostasis.

### KROX20 regulates Notch1 to control differentiation of SG resident stem cells.

*Notch1* signaling has been shown to directly promote differentiation of SG stem cells (9), and the context-dependent *Notch1* ligand JAG2 serves as the ligand establishing the *Jag2/Notch1* axis that governs SG stem cell differentiation (26). However, the gatekeeper mechanisms that balance *Notch1*-mediated differentiation and self-renewal in SG morphogenesis and homeostasis remain largely unknown. To understand the mechanism in SG stem cells, we investigated the expression of *Jag2* and *Notch1* in SGs: scRNA-seq analysis revealed high expression of both *Jag2* and *Notch1* mRNAs at the junction area (SG stem cell niche) between upper hair follicle cells and SG basal cells (7), suggesting the bifurcation signal leading to self-renewal or differentiation was located in SG stem cells (Figure 6A). Given KROX20^+^ cells are scarce in mouse skin and conventional ChIP-seq requires millions of cells, we employed CUT&RUN-seq, which requires far fewer cells (typically 1,000–100,000), making it well suited to identify KROX20-targeted genes in rare stem cell populations (34, 35). To improve peak resolution and confidence, 2 types of negative controls were included: CUT&RUN samples from wild-type mice (input) and *Krox20-cKO* mice, which lack KROX20. KROX20 binding peaks were called by comparing wild-type mice (pulldown with anti-KROX20 antibody) against either wild-type mice (input) or *Krox20-cKO* mice (pulldown with anti-KROX20 antibody). CUT&RUN-seq data confirmed that KROX20 directly binds the promotor of the *Notch1* gene, with the binding site located within 1 kb of the transcription start site (Figure 6B).

Further proof of KROX20 involvement in the regulation of *Notch1* signaling came from *Krox20-cKO* mice lacking KROX20 protein (Figure 2, B and C). HES1, a canonical downstream target of *Notch1* signaling whose expression reflects activation of *Notch1* signaling (36, 37), and Notch1/NTM were significantly downregulated in *Krox20-cKO* mice compared with littermates (Figure 6C and Supplemental Figure 8A). This indicated that absence of the *Krox20* gene/protein leads to inactivation of *Notch1* signaling by turning off *Notch1* transcription, confirming that KROX20 positively regulates *Notch1* in SG resident stem cells.

We next conditionally deleted *Notch1* in *Krox20*-expressing cells (*Notch1^fl/fl^ Krox20-Cre*; abbreviated as *Notch1-cKO*) (Figure 6D). Compared with 9M littermates, 9M *Notch1-cKO* mice exhibited an alopecia phenotype (Figure 6E). Whole-mount images revealed that hair follicles in tail skin of the *Notch1-cKO* mice ectopically developed multiple, atrophied SG lobes, with some lobes completely lacking lipid-producing sebocytes (Figure 6F; Supplemental Figure 8, B–H; and Supplemental Videos 7 and 8). Compared with 9M littermates, the lack of HES1 expression in tail and dorsal skin SGs of 9M *Notch1-cKO* mice validated the inactivation of *Notch1* signaling in *Krox20*-expressing SG stem cells (Supplemental Figure 8I). These atrophied SG lobes due to *Notch1* knockout aligned with the established role of *Notch1* signaling in promoting SG stem cell differentiation (9), as loss of this differentiation signal inhibits differentiation of SG stem cells, leading to a reduced number of downstream progenitors and sebocytes. The underlying mechanism of why ectopic atrophied SG lobes develop is not known. Double-staining of SG stem cell marker KROX20 and SG stem/progenitor marker LRIG1 showed that the percentage of KROX20^+^LRIG1^+^ cells was significantly higher in the SGs of 9M *Notch1-cKO* mice compared with littermates (Figure 6, G and H). We also observed ectopic expression of KROX20 in the entire atrophic SG lobes of *Notch1-cKO* mice, whereas KROX20 expression was normally restricted to the SG root area in littermates (Figure 6G). We therefore speculated that mice attempt to compensate for the functional decline caused by atrophic SG lobes due to loss of *Notch1* signaling by generating ectopic KROX20^+^ SG stem cells to form additional SG lobes in *Notch1-cKO* mice. However, with the *Notch1* differentiation signaling constitutively absent, these newly formed SG lobes remain underdeveloped (Figure 6I).

In addition to using the constitutive *Notch1*-knockout system to determine the effect on SG morphogenesis, we generated an inducible *Notch1*-knockout model (*Notch1^fl/fl^ Krox20-CreERT2*; abbreviated as *Notch1-iKO* mice) to determine the effect of *Notch1* deletion during SG homeostasis (Supplemental Figure 9A). In line with the results from the noninducible *Notch1*-knockout system, whole-mount visualization of tail SGs revealed reduced SG size in *Notch1-iKO* mice (Supplemental Figure 9, B–E).

Together, these results demonstrate that KROX20 is essential not only for maintaining the self-renewal of SG resident stem cells but also for directing their differentiation through direct control of the *Notch1* gene.

### Notch1 ectopic expression rescues SG loss caused by deletion of Krox20.

Additional evidence supporting the link between KROX20 and *Notch1* signaling came from in vivo rescue experiments. We found that loss of the *Krox20* gene and its encoded KROX20 protein suppressed *Notch1* transcription, resulting in atrophied SG lobes in *Krox20-cKO* mice. Therefore, we asked whether overexpression of the Notch1 intracellular domain (NICD) could reactivate *Notch1* signaling and restore differentiation cues in SG resident stem cells, thereby rescuing the SG morphology phenotype in *Krox20-cKO* mice. To test this, we used *ACTB-NICD* mice, in which the NICD is driven by the β-actin promoter and conditionally overexpressed upon Cre recombinase activity (38). We generated *Krox20^fl/fl^ K14-Cre ACTB-NICD* (*Krox20-cKO Notch1-OE*) mice and euthanized them at 4 months of age to analyze SG morphology (Figure 7A). Whole-mount visualization of tail skin SGs revealed that *Notch1* overexpression in *Krox20-cKO* mice rescued the SG loss caused by *Krox20* deficiency (Figure 7B, Supplemental Figure 10, A and B, and Supplemental Videos 9–11). In *Krox20-cKO Notch1-OE* mice, the percentage of hair follicles with SGs (Figure 7C); the distribution of 0-, 1-, and 2-SG (Figure 7D); the average number of SG lobes per hair follicle (Figure 7E); and the average length (Figure 7F) and average area (Figure 7G) of SG lobes were all restored compared with *Krox20-cKO* mice. Furthermore, assessment of SG lipid production (Supplemental Figure 10C) along with measurements of TEWL and TEHL (Figure 7H) additionally suggested that the rescued SGs in *Krox20-cKO Notch1-OE* mice exhibited rescued sebum production compared with *Krox20-cKO* mice. Double-staining of KROX20 and LRIG1 showed that the percentage of LRIG1^+^ cells per gland was rescued to wild-type levels in *Krox20-cKO Notch1-OE* mice compared to *Krox20-cKO* mice (Supplemental Figure 10D), suggesting that overexpression of *Notch1* signaling, marked by HES1 overexpression, rescued the atrophied SG lobe phenotype in *Krox20-cKO* mice by restoring the early differentiation process from SG stem cells into progenitors and subsequent differentiation of progenitors into differentiating PPARγ^+^ sebocytes (Supplemental Figure 10E).

To complement these genetic *Notch1*-overexpression studies, we also tested whether pharmacological activation of *Notch1* signaling could rescue the atrophied SG lobes resulting from inducible *Krox20* loss in *Krox20^fl/fl^ K14-CreERT2* (*Krox20-iKO*) mice. Valproic acid (VPA) has been shown to activate *Notch1* signaling by inhibiting histone deacetylases and upregulating *Notch1* expression (39–41). We therefore topically treated *Krox20-iKO* mice with VPA (500 mM) on dorsal and tail skin daily for 7 days, along with the induction with 4 mg tamoxifen starting at 5.5 months of age (Supplemental Figure 11A). Compared with *Krox20-iKO* mock-treated mice, the SG phenotype in dorsal and tail skin from VPA-treated *Krox20-iKO* mice was rescued (Supplemental Figure 11, B–D), supporting that *Notch1* signaling acts downstream of *Krox20* in the SGs.

### Topical Notch1 signaling modulation demonstrates potential for maintaining skin oil balance.

Based on the above results, we speculated that enhanced *Notch1* signaling would promote differentiation of SG stem cells into progenitors and sebocytes, resulting in an oily skin phenotype, whereas inhibition of *Notch1* signaling would suppress this differentiation program, leading to dry skin. As a proof-of-concept experiment, we analyzed SGs in 6-month-old *K14-Cre* *ACTB-NICD* (*Notch1-OE*) mice (Figure 8A). RT-qPCR of epidermis from *Notch1-OE* mice showed increased transcription of *Hes1* compared with littermates, confirming *Notch1* overexpression (Figure 8B). Compared with littermates, dorsal and tail skin SGs of *Notch1-OE* mice were larger (Figure 8, C–H), with potentially enhanced lipid film–based barrier protection (Figure 8, I and J). Together, SG enlargement driven by enhanced *Notch1* signaling (Figure 8, A–J) and SG atrophy resulting from *Notch1* knockout (Figure 6F) provide proof of concept for our initial speculation that regulating skin dryness and oiliness can be achieved through modulation of *Notch1* signaling.

Next, we tested *Notch1* signaling activator VPA (39–41) and inhibitor DAPT (42, 43) in wild-type mice. Mice were mock-, DAPT-, or VPA-treated for 14 days starting at P25 (Figure 8K), and then the SGs were analyzed. We observed atrophied SG lobes in the DAPT-treated cohort and enlarged SG lobes in the VPA-treated cohort compared with the mock-treated cohort (Figure 8, L–N). HES1 expression was, as expected, downregulated in DAPT-treated mice and upregulated in VPA-treated mice (Supplemental Figure 12). Importantly, TEWL was significantly higher in the DAPT-treated cohort compared with the mock-treated cohort (Figure 8O). Conversely, VPA treatment lowered TEWL to levels below those of the mock-treated cohort, suggesting increased sebum production (Figure 8O). Overall, these topical manipulations of *Notch1* signaling using small molecules suggested that skin oiliness and dryness can be modulated through the *Notch1* pathway. This finding provides proof of concept that modulating other signaling pathways that act downstream of KROX20 could be targets for treatment of skin conditions caused by SG dysfunction, such as dry skin, atopic dermatitis, and acne vulgaris.

## Discussion

Although MGs and SGs are both holocrine lipid-secreting glands, SGs are anatomically connected to hair follicles, forming the pilosebaceous unit, whereas MGs are not associated with hair follicles. Consequently, SGs and hair follicles constitute an integrated, functionally, and biologically interdependent unit (44, 45). The critical role of the pilosebaceous unit in maintaining skin homeostasis has prompted us to further investigate SGs and their resident stem cells. Although a few SG stem cell markers, such as LRIG1 (12–14) and GATA6 (15, 16), have been reported, the underlying molecular mechanisms regulating self-renewal and differentiation of SG stem cells are still not well understood. In this study, we have demonstrated that KROX20 is a SG resident stem cell marker that plays a crucial role in maintaining SG resident stem cells. We also showed that KROX20 regulates *Notch1* signaling, governing the differentiation of SG stem cells (Supplemental Figure 13). Furthermore, we generated an inducible KROX20^+^ cell ablation mouse model (*Krox20-GFP-DTA K14-CreERT2*) that rapidly and robustly develops systemic dry, flaky, alopecic skin upon tamoxifen induction. This model, termed xeroflacia, represents a platform for studying the biology of dry skin.

The Asebia mouse, identified as a spontaneous alopecic mutant lacking SGs, was described over 60 years ago in 1965 (1), and the Asebia mutation was subsequently traced to deletion of *Scd1*, a gene encoding stearoyl-CoA desaturase 1 (SCD1) acting as a rate-limiting enzyme for monounsaturated fatty acid synthesis, in 1999 (46). However, SGs in Asebia mice are hypoplastic rather than aplastic since the deletion of *Scd1* results in the differentiating and differentiated sebocytes degenerating within the SGs (1, 46). With the emergence of new insights into SG stem cells, we have identified KROX20 as a marker of SG stem cells. Ablation of KROX20^+^ SG resident stem cells in xeroflacia mice eliminates SGs by targeting the upstream stem cell reservoir, resulting in a cascade of events, including systemic loss of SGs and compromised barrier function. This predisposes the skin to theoretical pathogen invasion and immune cell infiltration. This differs from the Asebia model, which affects downstream sebocytes at later differentiation stages. Furthermore, specifically ablating SG resident stem cells in a finely controlled manner during hair follicle and SG morphogenesis and homeostasis enables mechanistic analysis of immune regulation. There is pronounced loss of dWAT and dermal collagen/fibronectin in the xeroflacia model as well, and we speculate that these phenotypes may result from altered epithelial-mesenchymal crosstalk following ablation of KROX20^+^ cells. SGs and hair follicles are known to interact closely with surrounding dermal adipocytes and fibroblasts through paracrine signaling pathways that regulate extracellular matrix deposition and tissue remodeling (47, 48). Therefore, degeneration of SGs and associated hair follicle abnormalities may secondarily impair maintenance of dWAT and dermal matrix integrity. In addition, immune alterations, particularly the pronounced macrophage infiltration observed in the xeroflacia model, may further contribute to loss of dWAT, as macrophage-adipocyte interaction has been reported to stimulate adipocyte lipolysis (33). The reduction in dWAT and dermal collagen/fibronectin is consistent with the weight loss and decreased body size observed in xeroflacia mice (Figure 4B).

We have shown that the KROX20^+^ cells not only function as SG resident stem cells (this study) but also can contribute to the IFE (18) and serve as ancestors of hair follicle bulge stem cells (17) within the epidermal compartment. The multilineage contribution capacity of KROX20-positive cells suggests cellular heterogeneity, implying that ablation of these cells not only leads to SG loss but also disrupts IFE homeostasis. Therefore, the resulting barrier dysfunction in the xeroflacia model is likely caused by multiple contributing factors.

We identified KROX20^+^ SG resident stem cells as indispensable for SG morphogenesis and homeostasis. Ablation of KROX20^+^ cells led to complete loss of SGs in dorsal skin, suggesting that KROX20 marks the majority of, if not all, SG resident stem cells (Figure 4C). However, we cannot entirely exclude the possibility that a minor subset of KROX20-negative SG resident stem cells exists, as ablation of KROX20^+^ cells in tail skin results in substantial SG atrophy rather than complete SG loss (Figure 4F).

Stem cell compartments within the pilosebaceous unit, such as SG resident stem cells and hair follicle bulge stem cells, have been reported to be distinct compartments, since upper and lower compartments of the pilosebaceous unit are maintained in autonomous manners (10, 11). However, recent reports suggested that these stem cell compartments are not completely independent but rather exhibit a dynamic “flow” within the pilosebaceous unit during wound healing (7, 49). Therefore, cells from non-SG compartments may contribute to the SG stem cell niche and begin expressing KROX20 under certain conditions.

One limitation of our study is that sebum production was evaluated using indirect measures, including TEWL/TEHL and TLC-based lipid profiling, rather than direct measurement of sebum secretion. Thus, these findings provide indirect evidence of altered SG function and sebum production. Another limitation of this study is the relatively small sample size for the drug treatment experiments, with 3 independent mice included in each group. A larger sample size with balanced representation of both sexes would help determine whether sex is a potential confounding variable and improve the generalizability of these findings. Nonetheless, our studies support the role of KROX20 as a regulator of SG stem cell biology, maintaining stemness and directing differentiation through regulation of *Notch1*.

## Methods

### Sex as a biological variable.

Our study examined male and female animals, and similar findings are reported for both sexes.

### Animal models.

Mice were housed under a 12-hour light/12-hour dark cycle at 21°C–23°C and a humidity of 40%–60%. *K14-Cre* (50) (strain 018964; RRID:IMSR_JAX:018964), *K14-CreERT2* (31) (strain 005107; RRID:IMSR_JAX:005107), *R26-tdTomato* (29) (strain 007914; RRID:IMSR_JAX:007914), *Notch1-flox* (38) (strain 006951; RRID:IMSR_JAX:006951) and *ACTB-NICD* (38) (strain 006481; RRID:IMSR_JAX:006481) mice were purchased from The Jackson Laboratory. The *Krox20-Cre* and *Krox20-flox* mouse lines were provided by Piotr Topilko (Institut Mondor de Recherche Biomédicale, Créteil, France) (28, 51). The *Krox20-flox-eGFP-flox-DTA* knockin allele mouse line was a gift from Piotr Topilko (Institut Mondor de Recherche Biomédicale, Créteil, France) and Patrick Charnay (Institut de Biologie de l’École Normale Supérieure, Paris, France) (30). *Krox20-CreERT2* and *R26-Krox20* mice were generated by the Mouse Genome Engineering Facility at UT Southwestern’s Children’s Research Institute.

### Whole-mount SG visualization in dorsal skin.

A 1 × 1 cm section of depilated dorsal skin was excised, flattened onto a SYLGARD 184 Silicone Elastomer-coated Corning 100 mm × 20 mm Style Dish, and immersed in 1 × PBS, and the hypodermis was finely removed under a dissecting microscope. Subsequently, the dorsal skin was stretched on a paper towel with the hair-bearing side facing upward, covered with No-Wrinkle Rubber Cement (Elmer’s) for 10 minutes, secured with Staples Invisible Tape, and then incubated overnight at 37°C in 5 mM EDTA/1 × PBS to weaken the epidermis-dermis adhesion. The next day, the quadruple structure (tape, cement, dorsal skin, and paper towel) was positioned under a dissecting microscope with the tape side facing downward. The dual structure (dermis and paper towel) was finely separated with fine tweezers, leaving the epidermis containing hair follicles and SGs adhered to the tape/cement, thereby forming the triple structure (tape, cement, and epidermis). This triple structure was fixed for 1 hour at room temperature in 4% paraformaldehyde (PFA)/1× PBS, washed with 1× PBS, and stained with Nile Red (50 μM, N1142, Invitrogen) for 30 minutes at room temperature in the dark. The stained triple structure was positioned onto Superfrost glass slides with the epidermis side facing downward and mounted in DAPI Fluoromount-G (0100-20, SouthernBiotech) for counterstaining cellular nuclei (9).

### Whole-mount SG visualization in tail skin.

The depilated tail was circumferentially cut at its base, followed by a longitudinal incision along the ventral surface extending the length of the tail. The tail skin was peeled off, cut into ~0.2 × 0.2 cm segments, and incubated overnight at 37°C in 5 mM EDTA/1× PBS to weaken the epidermis-dermis adhesion. The next day, the epidermis containing hair follicles and SGs were separated from the dermis under a dissecting microscope, and the separated epidermis was fixed for 1 hour at room temperature in 4% PFA/1× PBS, washed with 1× PBS, and stained with Nile Red (50 μM, N1142, Invitrogen) for 30 minutes at room temperature in the dark. The stained epidermis was positioned onto Superfrost glass slides with the pilosebaceous unit side facing downward, then mounted in DAPI Fluoromount-G (0100-20, SouthernBiotech) for counterstaining cellular nuclei (52).

Fluorescence images were acquired using an Olympus IX73 fluorescence microscope equipped with cellSens Standard software (Version 1.8) or Leica DMi8 confocal microscope with LAS AF software (Version 3.0). SG morphology parameters were measured using ImageJ (https://imagej.nih.gov/ij/). 3D construction was performed using Fiji (https://imagej.net/software/fiji/).

### Isolation of hair lipids and TLC analysis.

A 2.5 × 5 cm area of dorsal hair was collected from each mouse. Hair was weighed to ensure equal starting material across samples. A volume of 4 mL of chloroform-methanol (RSOC0020-1C, Ricca and UN1230, Alfa Aesar) (2:1, v/v) was added to each sample and incubated at room temperature overnight with rotation. The next day, 4 mL of acetone (179973, Sigma) was added to each sample for 10 minutes with rotation. Samples were filtered to remove hair and then left to vacuum evaporate until there was about 25 μL of sample left. An equal amount of chloroform-methanol (4:1, v/v) was added to the samples, pipetting the sides of the tube to get all lipid extracts. The standard control, nonpolar Lipid Mixture 1 (29376, Cayman), was added to the TLC plate (1003900001, Sigma) along with the lipid extractions (5 μL). The plate was placed in the first solvent mixture of hexane (296090, Sigma):isopropyl diether (673803, Sigma):acetic acid (179973, Sigma) (80:20:1, v/v) up to 50% of the plate height. The plate was removed and left to air-dry before being placed in the second solvent mixture of hexane/benzene (401765, Sigma) (1:1, v/v) up to 80% of the plate height. The plate was removed and dried before running in the third solvent mixture of hexane up to 90% of the plate height. The plate was removed and dried, then uniformly sprayed with a 10% cupric sulfate (451657, Sigma) in 8% phosphoric acid (695017, Sigma) solution and baked at 120°C for 20 minutes to visualize the lipid bands (53, 54). The area and intensity of bands were quantified using ImageJ (https://imagej.nih.gov/ij/).

### TEWL and TEHL measurement.

Mouse telogen dorsal skin was depilated 2 days before measurements. Mice were anesthetized with isoflurane, and the Tewameter TM Hex probe-MPA2 adaptor and MPA CTplus software (Courage-Khazaka Electronic) were used to measure TEWL and TEHL. Since a stable measurement is achieved within 30 seconds by the Tewameter TM Hex probe-MPA2 adaptor system, each measurement stops automatically at 30 seconds (45, 55).

### RNA extraction from mouse epidermis.

Dorsal skin in telogen was depilated and excised, then incubated overnight in 0.25% trypsin at 4°C. The next day, the epidermis containing hair follicles and SGs was separated from the dermis, then homogenized in TRI Reagent (T9424, Sigma-Aldrich). Total RNA was isolated following the TRIzol-chloroform extraction protocol (9, 56).

### RT-qPCR.

Reverse transcription was performed with the iScript cDNA Synthesis Kit (1708891, Bio-Rad). qPCR was conducted with PowerUp SYBR Green Master Mix (A25742, Applied Biosystems) and the QuantStudio 3 system (Applied Biosystems). The relative amounts of the amplified nucleotide fragments were calculated by the 2^(-ΔCt)^ method. Mouse *Eef1a1* and *Actb* were used as housekeeping genes.

Primer sequences for qPCR were Mouse *Hes1*: For: TCAACACGACACCGGACAAAC, Rev: ATGCCGGGAGCTATCTTTCTT; Mouse *Eef1a1*: For: CAACATCGTCGTAATCGGACA, Rev: GTCTAAGACCCAGGCGTACTT; Mouse *Actb*: For: GGCTGTATTCCCCTCCATCG, Rev: CCAGTTGGTAACAATGCCATGT.

### Induction with tamoxifen or 4-HT.

Tamoxifen was dissolved in corn oil by vortexing for 2 hours at room temperature at 20 mg/mL. A volume of 200 μL tamoxifen solution (4 mg) was given by gavage to adult mice. 4-HT was dissolved in alcohol at 5 mg/mL. Pups were subcutaneously injected with 10 μL 4-HT solution (50 μg) at the tail junction for perinatal induction.

### Immunofluorescence and H&E staining.

Dorsal or tail skin was removed, and the excised tissue was fixed in 10% formalin for 4 hours and processed by the Citadel 2000 Tissue Processor. The skin was then embedded in paraffin and sectioned at 5 μm thickness. The skin sections were then placed on Superfrost glass slides. For immunofluorescence staining, slides were deparaffinized and rehydrated, followed by antigen retrieval. The slides were then blocked with 10% donkey serum and incubated with primary antibodies, including Rabbit anti-EGR2 (KROX20) (PA5-27814, Thermo Fisher Scientific, RRID:AB_2545290, 1:150), Rabbit anti-RFP (600-401-379, Rockland, RRID:AB_2209751, 1:300), Rabbit anti-PPARγ (2435, Cell Signaling Technology, RRID:AB_2166051, 1:200), Mouse anti-FASN (sc-48357, Santa Cruz Biotechnology, RRID:AB_627584, 1:50), anti-Cytokeratin 14 (LL002) (Alexa Fluor 488) (NBP2-34675AF488, Novus, RRID:AB_3290990, 1:200), Goat anti-LRIG1 (AF3688-SP, Novus Biologicals, RRID:AB_ 2138836, 1:100), Rabbit anti-Ki67 (ab15580, Abcam, RRID:AB_443209, 1:100), Rabbit anti-CD3 (ab5690-100, Abcam, RRID:AB_305055, 1:100), Rabbit anti-CD4 (ab183685, Abcam, RRID:AB_2686917, 1:100), Rabbit anti-CD8 (PA5-81344, Thermo Fisher Scientific, AB_2788560, 1:200), Rat anti-F4/80 (ab6640, Abcam, RRID:AB_1140040, 1:200), Rabbit anti-iNOS (ab15323, Abcam, RRID:AB_301857, 1:100), Rabbit anti-CD206 (ab64693, Abcam, RRID:AB_1523910, 1:200), Rabbit anti-Collagen I (ab21286, Abcam, RRID:AB_446161, 1:200), anti-Fibronectin (Alexa Fluor 594) (ab309604, Abcam, RRID:AB_3086599, 1:200), Rabbit anti-HES1 (11988, Cell Signaling Technology, RRID:AB_2728766, 1:100), and Rabbit anti-Notch1/NTM (3608, Cell Signaling Technology, RRID:AB_2153354, 1:100) overnight at 4°C. Subsequently, the slides were incubated in secondary antibody conjugated with Cy3 AffiniPure Donkey Anti-Rabbit IgG (711-165-152, Jackson ImmunoResearch), Alexa Fluor 647 AffiniPure Donkey Anti-Rabbit IgG (711-605-152, Jackson ImmunoResearch), Cy3 AffiniPure Donkey Anti-Mouse IgG (715-165-150, Jackson ImmunoResearch), Cy3 AffiniPure Donkey Anti-Goat IgG (705-165-003, Jackson ImmunoResearch) or Cy3 AffiniPure Donkey Anti-Rat IgG (712-165-150, Jackson ImmunoResearch) for 2 hours at room temperature and mounted in DAPI Fluoromount-G (0100-20, SouthernBiotech) for counterstaining cellular nuclei. For H&E staining, after deparaffinization and rehydration, the slides were immersed in Gill 3 hematoxylin (72604, Thermo Fisher Scientific), followed by short washes with High Def (SL103, StatLab) and Bluing reagent (22-220-106, Thermo Fisher Scientific) and eosin-Y with phaloxine (71304, Thermo Fisher Scientific) following the manufacturer’s protocol (StatLab).

### CUT&RUN sequencing.

A 2 × 1.5 cm section of depilated telogen dorsal skin from wild-type and *Krox20-cKO* mice was excised, the hypodermis was removed under a dissecting microscope, and then the skin was finely minced using a razor blade on ice. The minced skin was lightly fixed using 0.1% formaldehyde for 2 minutes at room temperature, following termination of cross-linking using glycine. The fixed minced skin was then disaggregated into single-cell suspension using a Scilogex D160 Homogenizer. CUT&RUN was performed following the CUT&RUN Assay Kit instructions (86652, Cell Signaling Technology) (34, 35). Concanavalin A Bead–bound and digitonin-permeabilized cells were incubated using Rabbit anti-EGR2 (KROX20) (MA5-38053, Thermo Fisher Scientific, RRID:AB_2897971, 1:50) or Rabbit anti-Tri-Methyl-Histone H3 (Lys4) (C42D8) (9751, Cell Signaling Technology, RRID:AB_2616028, 2 μL per sample, positive control) at 4°C overnight. The DNA fragments were repaired, A-tailed, and further ligated with Illumina adapter. The final DNA library was then generated through size selection and PCR amplification. Raw sequencing reads were initially assessed for quality using fastQC (v 0.12.1), which reports metrics including per-base sequence quality, GC content, duplication levels, read length distribution, k-mer profiles, and potential adapter contamination. Adapter sequences and low-quality bases were subsequently removed using fastp (v 0.24.0) to generate high-quality clean reads. For fastp, the adapters of pair-end data can be trimmed automatically by per-read overlap analysis, which seeks the overlap of each pair of reads. The processed reads were aligned to the mouse reference genome (mm10) using bowtie2 (v 2.5.4) with the parameters “-I 25 -X 1000 --no-mixed --no-discordant --very-sensitive”, ensuring high-confidence, concordant paired-end alignments. Samtools (v 1.21) was then used for sorting, indexing, and mapping quality assessment. Mitochondrial reads, low-quality reads (Q < 30), unmapped reads, and multimapping reads were filtered out using samtools. Duplicates were marked using picard (v 3.4.0). Then, 2 types of visualization tracks (bigwig and bedgraph) were generated using deepTools (v 3.5.6). Peaks were called using Macs2 (v 2.2.6) with a significance threshold of *P* < 1 × 10^–5^. Differential binding site analysis was performed using DiffBind (v 3.16.0) with a significance threshold of *P* < 0.05. Peaks were subsequently annotated to their nearest genes using ChIPseeker (v 1.42.0) to facilitate biological interpretation.

### Notch1 pathway inhibition by DAPT treatment and activation by VPA treatment.

P25 wild-type mice were topically treated with 20 μL of DAPT (50 mM) or 20 μL of valproic acid sodium salt (VPA; 500 mM) or mock-treated (1:1 mixture of ethanol and PEG300) on the dorsal and tail skin daily for 14 consecutive days. DAPT (2634, Tocris) or VPA (P4543, Sigma) powder was dissolved in a 1:1 mixture of ethanol and PEG300 (v/v). 5.5-month-old *Krox20-iKO* mice were topically treated with 20 μL of VPA (500 mM) or mock-treated on the dorsal and tail skin daily for 7 consecutive days.

### Statistics.

Data represent mean ± SEM of ≥3 independent experiments. Statistical analyses were performed using unpaired 2-tailed *t* test, multiple unpaired *t* test, 2-way ANOVA with Tukey’s multiple comparisons test, 2-way ANOVA with Holm-Šídák multiple comparisons test, or 1-way ANOVA with Tukey’s multiple comparisons test by GraphPad Prism 10.

### Study approval.

Mouse care and experiments were approved by the Institutional Animal Care and Use Committee at University of Texas Southwestern Medical Center (protocol 2017-102108, PI LQL) and University of Virginia School of Medicine (protocol 4490, PI LQL).

### Data availability.

CUT&RUN-seq data were deposited in NCBI’s GEO database: GSE315331. All other data presented in this paper are provided in the Supporting Data Values file.

## Manuscript information

### Author contributions.

LQL and YZ conceived the project; YZ, RMM, and LQL planned the project; YZ, MFP, AL, and YH carried out the experiments; JY provided guidance on multiple experimental techniques; YZ and PP analyzed the scRNA-seq data; YZ generated the samples for CUT&RUN-seq and analyzed the CUT&RUN-seq data; YZ performed additional data analysis and prepared the figures; and YZ, RMM, and LQL wrote and revised the manuscript.

### AI disclosure.

During the preparation of this work, the authors used OpenAI’s ChatGPT to generate a mouse icon for the figures.

### Conflict of interest.

The authors have declared that no conflict of interest exists.

### Funding.

This study was supported by the following:

- NIH grant R01 EY033344 (to LQL).

This work is the result of NIH funding, in whole or in part, and is subject to the NIH Public Access Policy. Through acceptance of this federal funding, the NIH has been given a right to make the work publicly available in PubMed Central.

## Supplementary Material

Supplemental data

Supplemental video 1

Supplemental video 2

Supplemental video 3

Supplemental video 4

Supplemental video 5

Supplemental video 6

Supplemental video 7

Supplemental video 8

Supplemental video 9

Supplemental video 10

Supplemental video 11

Supporting data values

## Figures and Tables

**Figure 1:**
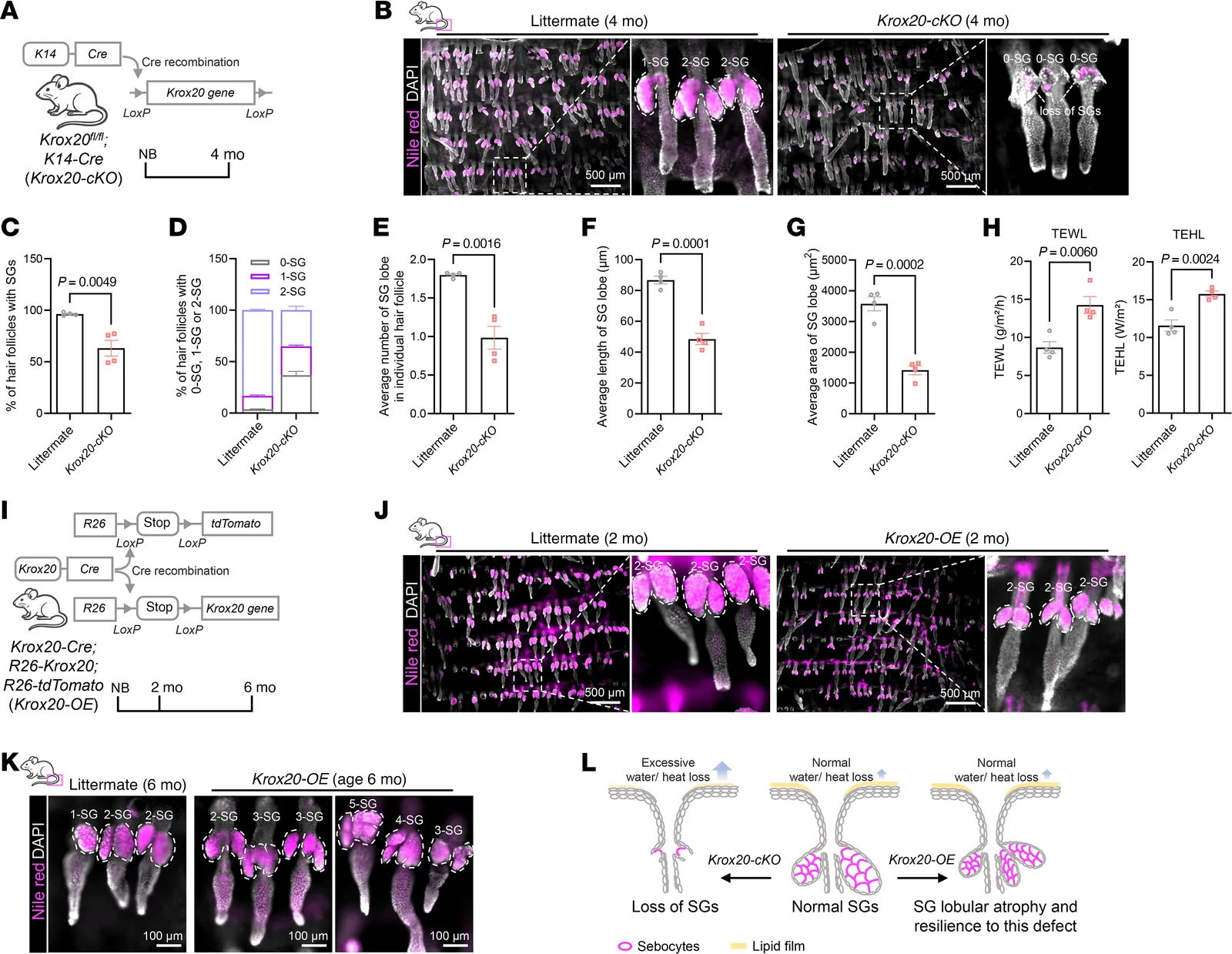
Deletion or overexpression of *Krox20* impairs SG morphogenesis. (**A**) Scheme for deleting *Krox20*; mice were sacrificed at 4 months of age (4M). *n* = 4 for littermate and *Krox20-cKO*. (**B**) Representative whole-mount visualization of tail skin SGs from littermate and *Krox20-cKO* mice. (**C**–**H**) Percentage of hair follicles with SGs (**C**); percentage of hair follicles with 0-, 1-, or 2-SG (**D**); average number of SG lobes in individual hair follicles (**E**); and average length (**F**) and average area (**G**) of SG lobes in tail skin and transepidermal water loss (TEWL) and transepidermal heat loss (TEHL) in dorsal skin (**H**) from littermate and *Krox20-cKO* mice. (**I**) Scheme for overexpressing *Krox20*; mice were sacrificed at 2M and 6M. *n* = 4 for littermate and *Krox20-OE* at each time point. (**J**) Representative whole-mount visualization of tail skin SGs from 2M mice. (**K**) Representative whole-mount visualization of tail skin SGs from 6M mice. (**L**) Schematic overview of SG morphology in *Krox20*-knockout, normal, and *Krox20*-overexpression scenarios. Two-way ANOVA with Holm-Šídák multiple comparisons test was used to calculate *P* value in **D**, comparisons between littermates and *Krox20-cKO* mice yielded the following *P* values: 0-SG, *P* < 0.0001; 1-SG, *P* = 0.0006; and 2-SG, *P* < 0.0001. Unpaired 2-tailed *t* test was used to calculate *P* values in **C** and **E**–**H**. (**A** and **I**) NB, newborn.

**Figure 2:**
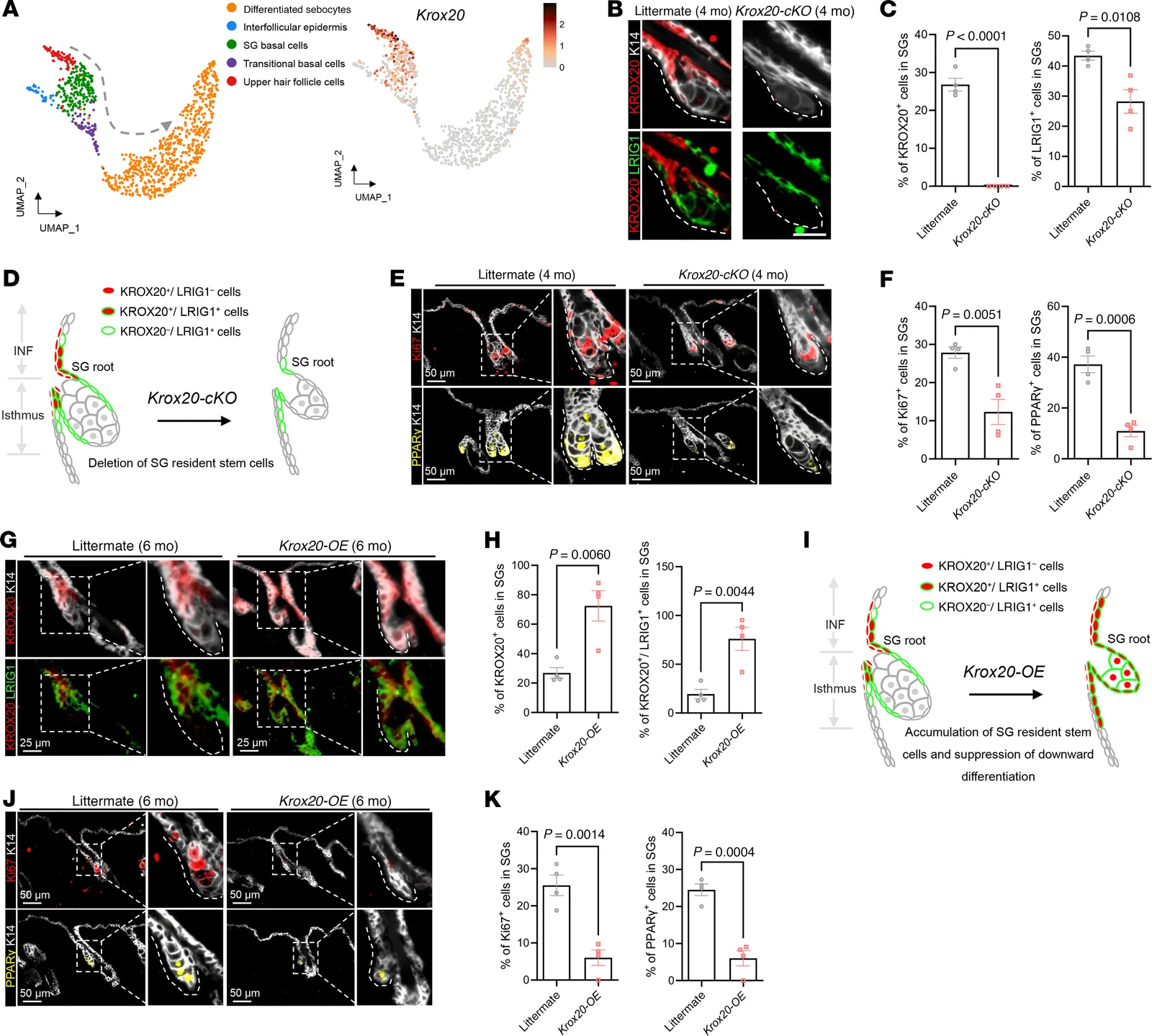
KROX20 controls cellular input to SGs by regulating SG resident stem cells. (**A**) Uniform manifold approximation and projection (UMAP) showing epidermal compartments (7). The gray dashed arrow denotes the differentiation direction from SG stem cells to differentiated sebocytes (left panel); UMAP showing *Krox20* expression in upper hair follicle cells and SG basal cells (right panel). (**B**) Paraffin sections of dorsal skin from littermate (4M) and *Krox20-cKO* (4M) mice, co-stained with anti-K14, anti-KROX20, and anti-LRIG1 antibodies. Scale bar = 25 μm. (**C**) Percentage of KROX20^+^ and LRIG1^+^ cells in SGs based on immunostaining data in **B**. (**D**) Schematic showing the distribution of KROX20^+^LRIG1^–^, KROX20^+^LRIG1^+^, and KROX20^–^LRIG1^+^ cells in dorsal skin SGs of littermate (4M) and *Krox20-cKO* (4M) mice. (**E**) Paraffin sections of dorsal skin from littermate (4M) and *Krox20-cKO* (4M) mice, costained with anti-K14/anti-Ki67 antibodies or anti-K14/anti-PPARγ antibodies. (**F**) Percentage of Ki67^+^ and PPARγ^+^ cells in SGs based on immunostaining data in **E**. (**G**) Paraffin sections of dorsal skin from littermate (6M) and *Krox20-OE* (6M) mice, costained with anti-K14, anti-KROX20, and anti-LRIG1 antibodies. (**H**) Percentage of KROX20^+^ and KROX20^+^LRIG1^+^ cells in SGs based on immunostaining data in **G**. (**I**) Schematic showing the distribution of KROX20^+^LRIG1^–^, KROX20^+^LRIG1^+^, and KROX20^–^LRIG1^+^ cells in dorsal skin SGs of littermate (6M) and *Krox20-OE* (6M) mice. (**J**) Paraffin sections of dorsal skin from littermate (6M) and *Krox20-OE* (6M) mice, costained with anti-K14/anti-Ki67 antibodies or anti-K14/anti-PPARγ antibodies. (**K**) Percentage of Ki67^+^ and PPARγ^+^ cells in SGs based on immunostaining data in **J**. Normal or atrophied SGs outlined by white dashed lines in **B**, **E**, **G**, and **J**. Unpaired 2-tailed *t* test was used to calculate *P* values in **C**, **F**, **H**, and **K**. Hair cycle was matched for quantifications in **B**, **C**, **E**–**H**, **J**, and **K**. (**D** and **I**) INF, infundibulum.

**Figure 3:**
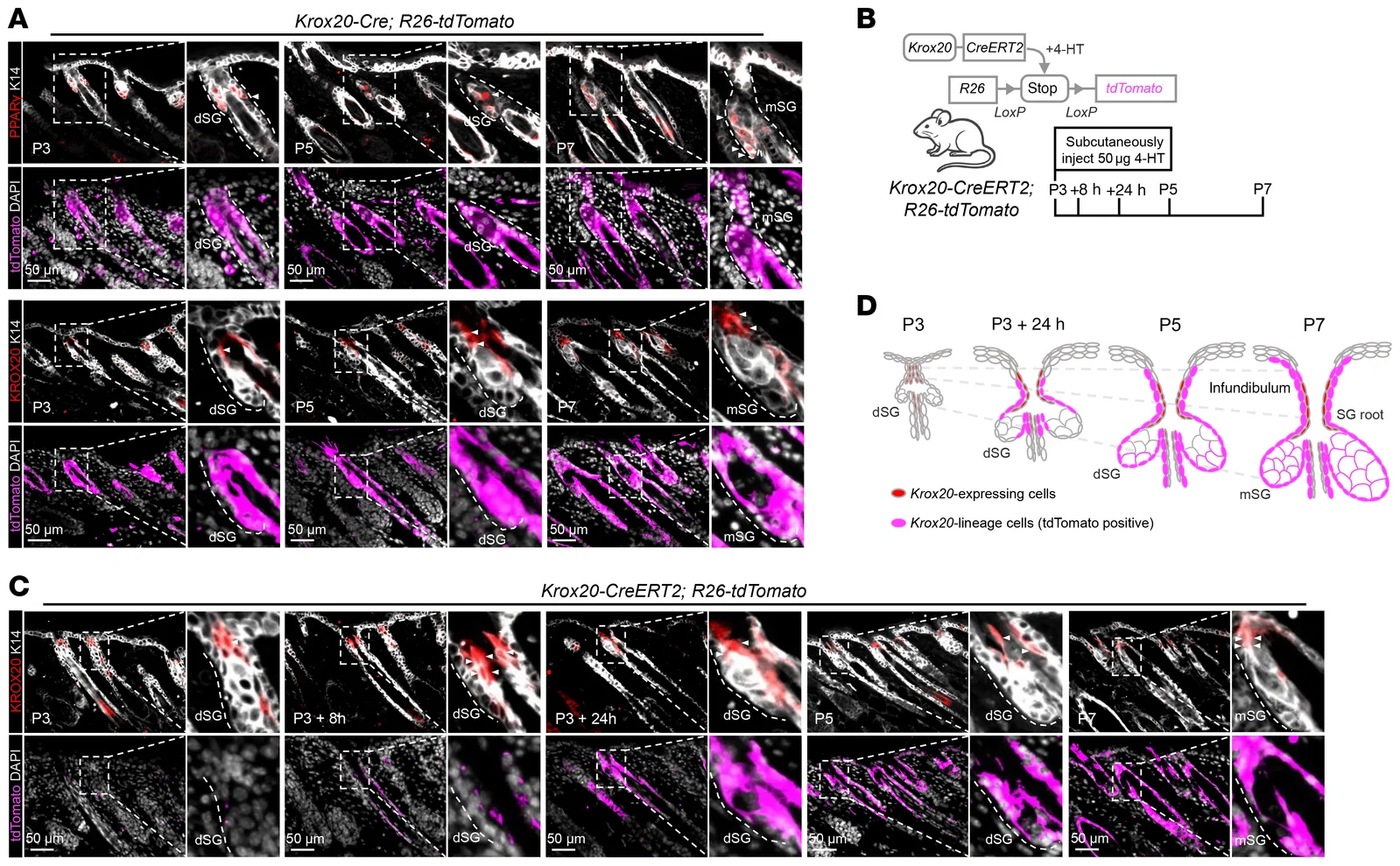
KROX20 marks SG stem cells, and *Krox20-*lineage cells contribute to SG morphogenesis. (**A**) Paraffin sections of dorsal skin from noninducible *Krox20-Cre R26-tdTomato* mice at P3, P5, and P7, co-stained with anti-K14, anti-PPARγ, and anti-tdTomato antibodies (upper panel) and anti-K14, anti-KROX20, and anti-tdTomato antibodies (lower panel). Developing SGs (dSG; before P7) or mature SGs (mSG; P7) demarcated with white dashed lines and PPARγ^+^ and KROX20^+^ cells indicated by white arrowheads. *n* = 3 for each time point. (**B**) Schematic illustrating *Krox20*-lineage tracing during SG morphogenesis in inducible *Krox20-CreERT2 R26-tdTomato* mice. Mice were induced at P3 with 50 μg 4-HT, then sacrificed at P3, P3 + 8h, P3 + 24h, P5, and P7. *n* = 3 for each time point. (**C**) Paraffin sections of dorsal skin from *Krox20-CreERT2 R26-tdTomato* mice, co-stained with anti-K14, anti-KROX20, and anti-tdTomato antibodies. KROX20^+^ cells indicated by white arrowheads. (**D**) Schematic depicting the developmental process of infundibulum and SGs from P3 to P7 with the distribution of *Krox20*-expressing cells and *Krox20*-lineage cells.

**Figure 4:**
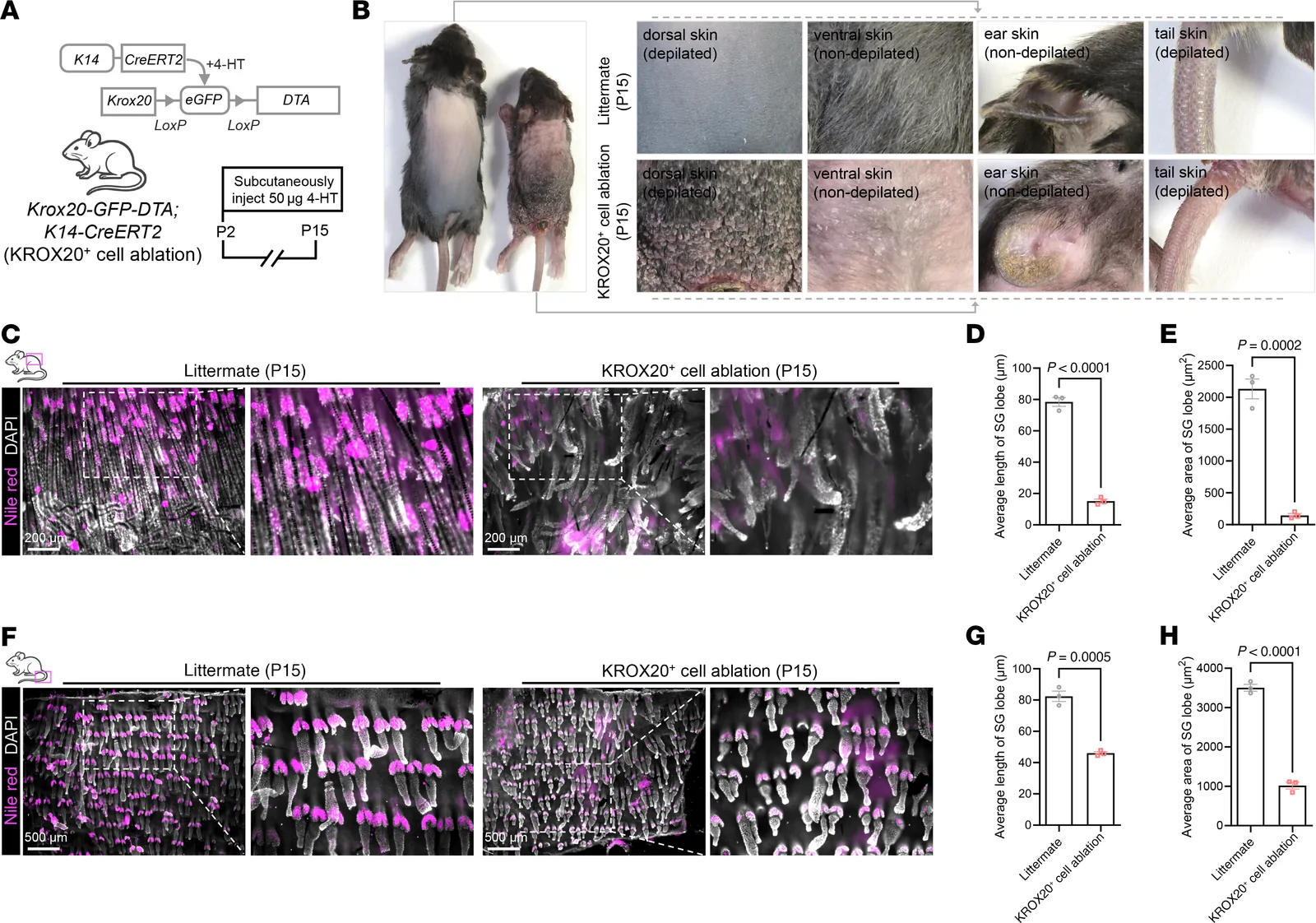
KROX20^+^ cell ablation depletes SGs, leading to dry, flaky skin. (**A**) Schematic showing ablation of KROX20^+^ cells during SG morphogenesis in *Krox20-GFP-DTA K14-CreERT2* (KROX20^+^ cell ablation) mice. Following induction at P2 with 50 μg 4-HT, mice were harvested at P15. *n* = 3 for each genotype. (**B**) Representative images of dorsal skin, ventral skin, ear skin, and tail skin from littermate and KROX20^+^ cell ablation mice. (**C**) Representative whole-mount dorsal skin images of SGs from littermates and KROX20^+^ cell ablation mice. (**D** and **E**) Average length (**D**) and average area (**E**) of SG lobes in dorsal skin. (**F**) Representative whole-mount tail skin images of SGs from littermate and KROX20^+^ cell ablation mice. (**G** and **H**) Average length (**G**) and average area (**H**) of SG lobes in tail skin. Unpaired 2-tailed *t* test was used to calculate *P* values in **D**, **E**, **G**, and **H**.

**Figure 5:**
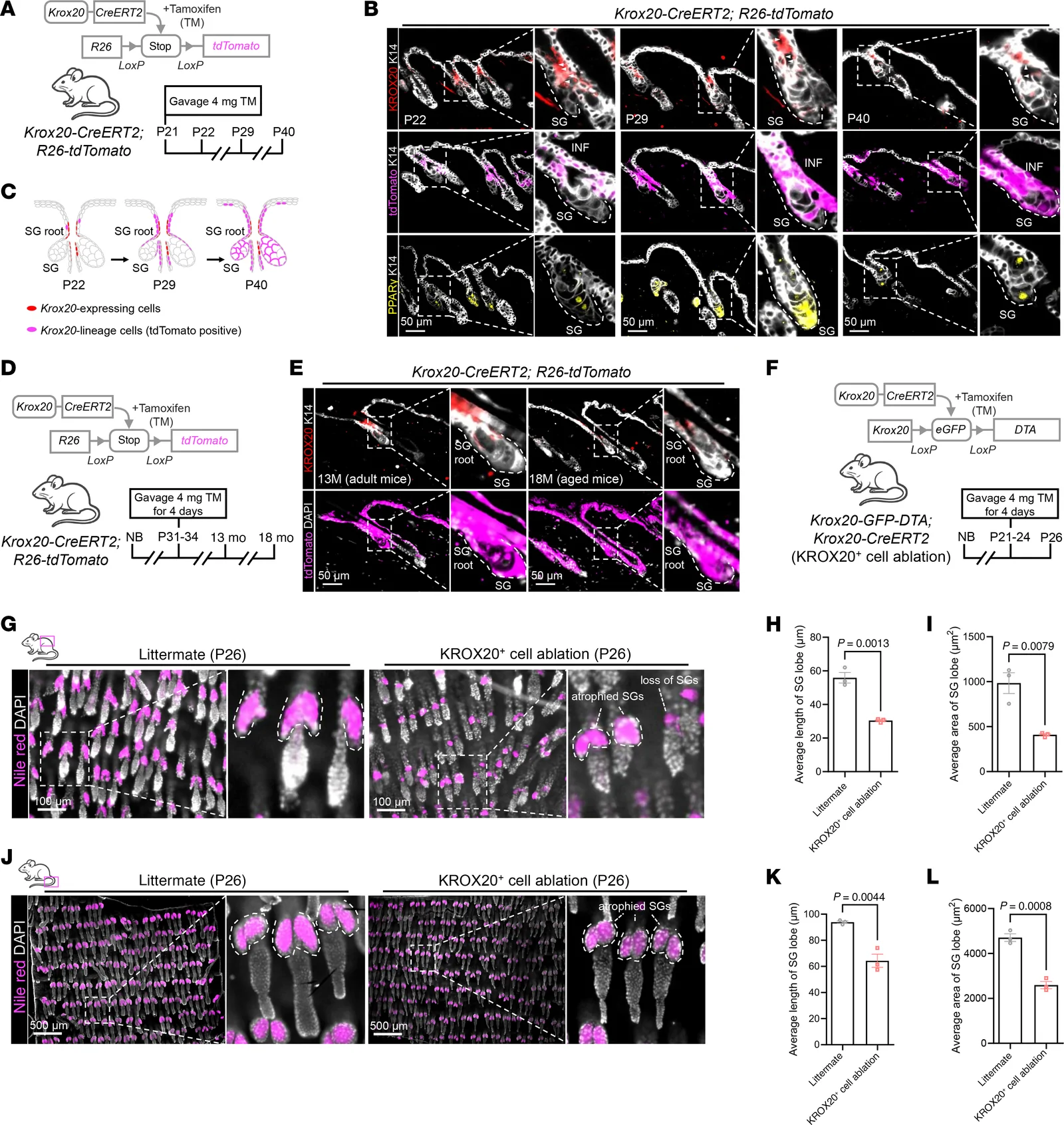
KROX20 marks SG stem cells, and *Krox20-*lineage cells contribute to SG homeostasis. (**A**) Schematic illustrating *Krox20*-lineage tracing during SG homeostasis in inducible *Krox20-CreERT2 R26-tdTomato* mice. Following induction at P21, mice were harvested at P22, P29, and P40. *n* = 3 for each time point. (**B**) Paraffin sections of dorsal skin from *Krox20-CreERT2; R26-tdTomato* mice, costained with anti-K14/anti-KROX20 antibodies, anti-K14/anti-tdTomato antibodies, and anti-K14/anti-PPARγ antibodies. SGs are demarcated with white dashed lines and KROX20^+^ cells are indicated by white arrowheads. (**C**) Schematic showing the distribution of *Krox20*-expressing cells and *Krox20*-lineage cells. (**D**) Schematic showing *Krox20*-lineage tracing in inducible *Krox20-CreERT2 R26-tdTomato* mice. Following induction at P31–P34, mice were harvested at 13M (adult mice) and 18M (aged mice), respectively. (**E**) Paraffin sections of dorsal skin from *Krox20-CreERT2 R26-tdTomato* mice at 13M and 18M, co-stained with anti-K14, anti-tdTomato, and anti-KROX20 antibodies. (**F**) Scheme for ablating KROX20^+^ cells during SG homeostasis in *Krox20-GFP-DTA Krox20-CreERT2* (KROX20^+^ cell ablation) mice. Following induction at P21–P24, mice were sacrificed at P26. *n* = 3 for each genotype. (**G**) Representative whole-mount dorsal skin images of SGs from littermate and KROX20*^+^* cell ablation mice. (**H** and **I**) Average length (**H**) and average area (**I**) of SG lobes in dorsal skin from littermate and KROX20^+^ cell ablation mice. (**J**) Representative whole-mount tail skin images of SGs from littermate and KROX20^+^ cell ablation mice. (**K** and **L**) Average length (**K**) and average area (**L**) of SG lobes in tail skin from littermate and KROX20^+^ cell ablation mice. Unpaired 2-tailed *t* test was used to calculate *P* values in **H**, **I**, **K**, and **L**.

**Figure 6:**
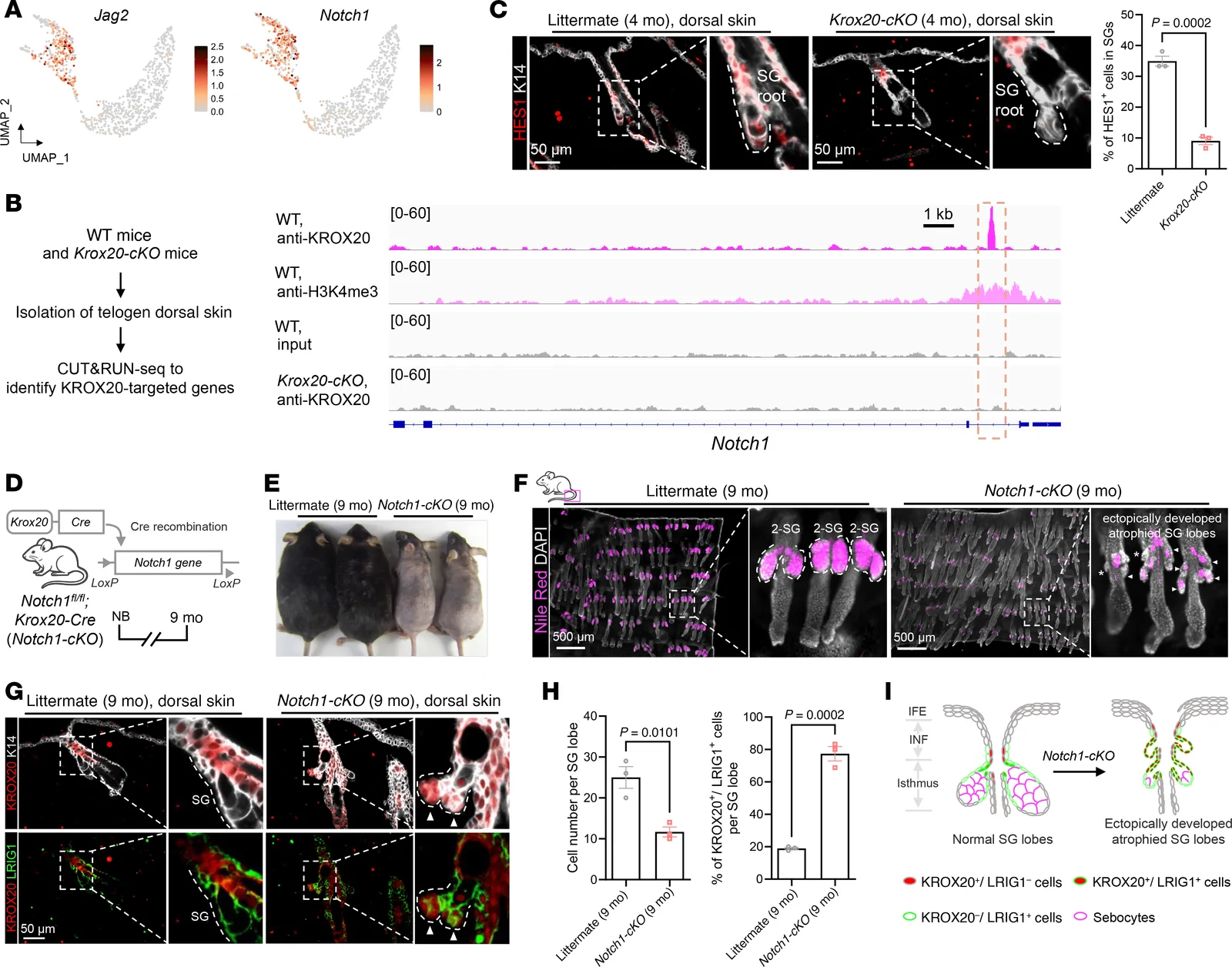
KROX20 regulates *Notch1* to control differentiation of SG resident stem cells. (**A**) UMAP showing the expression of *Jag2* and *Notch1*. (**B**) Workflow for performing CUT&RUN sequencing in wild-type (4M) and *Krox20-cKO* (4M) mice (left panel); KROX20 binds the *Notch1* gene (right panel). *n* = 3 for each genotype. (**C**) Paraffin sections of dorsal skin from littermate (4M) and *Krox20-cKO* (4M) mice, costained with anti-K14/anti-HES1 antibodies (left panel). Corresponding percentage of HES1^+^ cells in SGs (right panel). (**D**) Scheme for deleting *Notch1* in *Krox20*-expressing cells in *Notch1^fl/fl^ Krox20-Cre* (*Notch1-cKO*) mice, with mice analyzed at 9M. *n* = 3 for each genotype. (**E**) Whole-body images of mice without depilation. (**F**) Representative whole-mount tail skin images of SGs. Ectopically developed atrophied SG lobes are indicated with white arrowheads (with residual lipid-producing cells) and white asterisks (without lipid-producing cells). (**G**) Paraffin sections of dorsal skin, co-stained with anti-K14, anti-KROX20, and anti-LRIG1 antibodies. Ectopically developed atrophied SG lobes denoted with white arrowheads. (**H**) Quantification for cell number and percentage of KROX20^+^LRIG1^+^ cells per SG lobe based on immunostaining data in **G**. (**I**) Schematic showing ectopically developed atrophied SG lobes and the distribution of ectopic KROX20^+^/LRIG1^+^ cells in *Notch1-cKO* mice. Unpaired 2-tailed *t* test was used to calculate *P* values in **C** and **H**. Hair cycle was matched for quantifications in **C**, **G**, and **H**.

**Figure 7:**
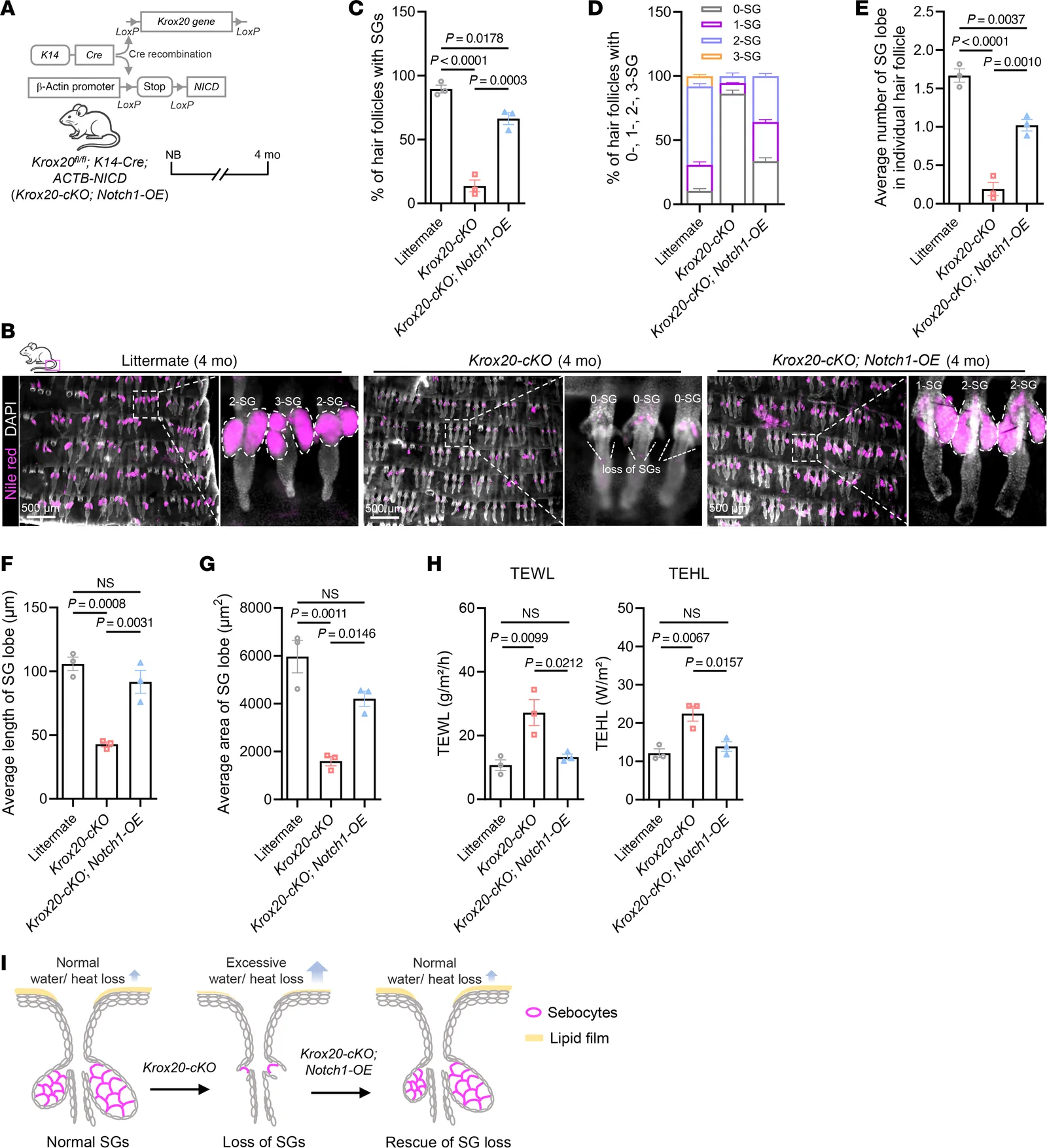
*Notch1* ectopic expression rescues SG loss caused by *Krox20* deletion. (**A**) Scheme for overexpressing the *NICD* in *Krox20^fl/fl^ K14-Cre ACTB-NICD* (*Krox20-cKO Notch1-OE*) mice. Mice were analyzed at 4M. *n* = 3 for each genotype. (**B**) Representative whole-mount tail skin images of SGs from littermate (4M), *Krox20-cKO* (4M), and *Krox20-cKO Notch1-OE* (4M) mice. (**C**–**G**) Percentage of hair follicles with SGs (**C**); percentage of hair follicles with 0-, 1-, 2-, or 3-SG (**D**); average number of SG lobes in individual hair follicles (**E**); and average length (**F**) and average area (**G**) of SG lobes in tail skin. (**H**) TEWL and TEHL in dorsal skin. (**I**) Diagram showing *Notch1* overexpression rescues SG loss and TEWL and TEHL caused by *Krox20* knockout. Two-way ANOVA with Tukey’s multiple comparisons test was used to calculate *P* value in **D**. Comparisons between littermates and *Krox20-cKO* mice yielded the following *P* values: 0-SG, *P* < 0.0001; 1-SG, *P* = 0.0004; and 2-SG, *P* < 0.0001. Comparisons between *Krox20-cKO* and *Krox20-cKO Notch1-OE* mice yielded the following *P* values: 0-SG, *P* < 0.0001; 1-SG, *P* < 0.0001; and 2-SG, *P* < 0.0001. One-way ANOVA with Tukey’s multiple comparisons test was used to calculate *P* values in **C** and **E**–**H**.

**Figure 8:**
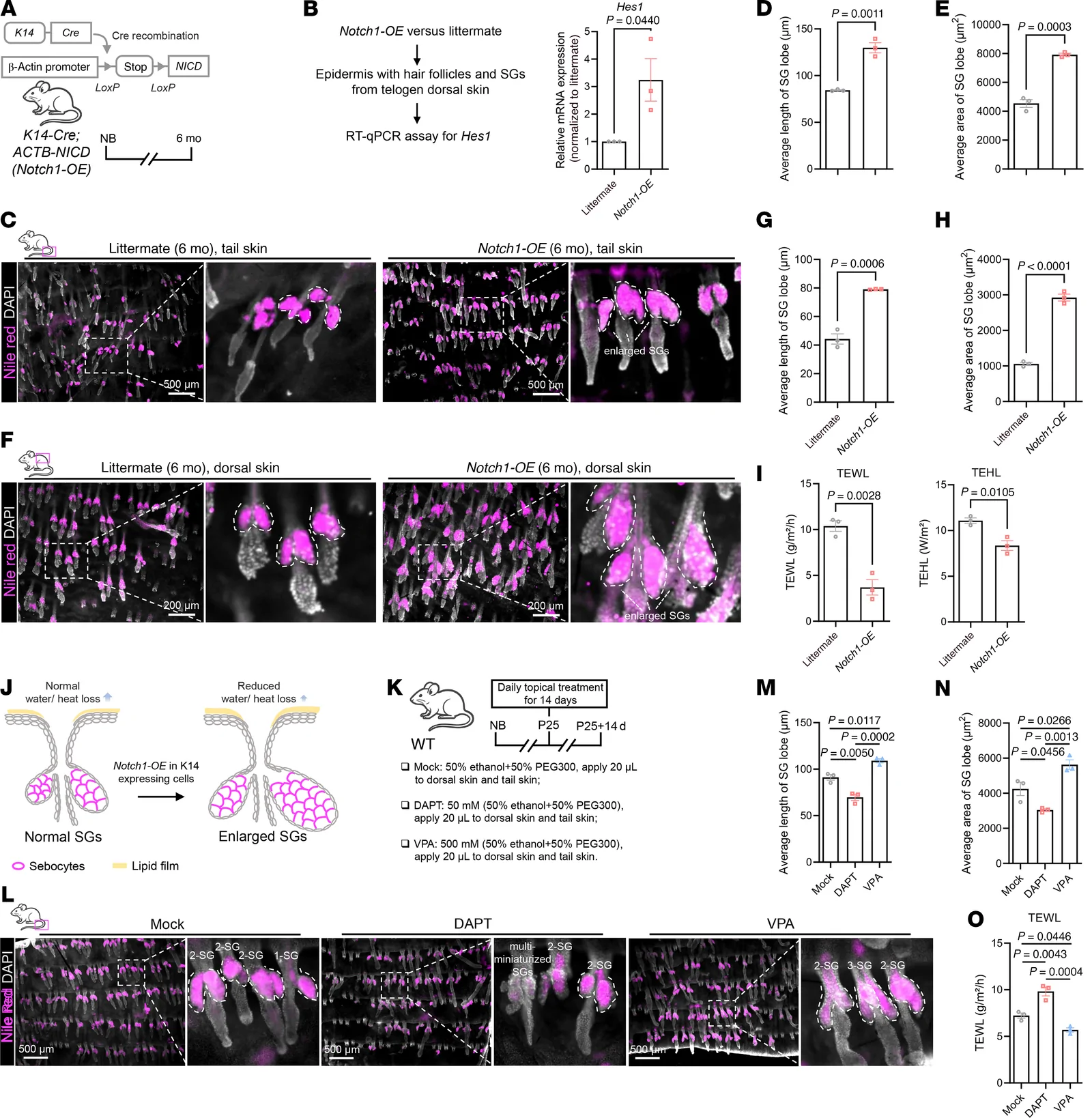
Genetic and pharmacological activation of *Notch1* results in enlarged SGs. (**A**) Schematic showing experimental strategy for overexpressing the *NICD* in *K14-Cre ACTB-NICD* (*Notch1-OE*) mice. Mice were analyzed at 6M. *n* = 3 for each genotype. (**B**) Workflow to collect epidermis to perform RT-qPCR assay (left panel); RT-qPCR assay of *Hes1* transcripts in dorsal skin from littermate (6M) and *Notch1-OE* (6M) mice (right panel). (**C**) Representative whole-mount images of tail skin SGs. (**D** and **E**) Average length (**D**) and average area (**E**) of SG lobes in tail skin. (**F**) Representative whole-mount dorsal skin images of SGs. (**G** and **H**) Average length (**G**) and average area (**H**) of SG lobes in dorsal skin. (**I**) TEWL and TEHL in dorsal skin. (**J**) Diagram showing SG lobe enlargement and decrease of TEWL and TEHL caused by genetic overexpression of *Notch1*. (**K**) Schematic illustrating the strategy for daily topical treatment of wild-type mice with mock, DAPT (Notch1 signaling inhibitor), and VPA (Notch1 signaling activator) from P25 for 14 days. *n* = 3 for each treatment cohort. (**L**) Representative whole-mount tail skin images of SGs from mock-, DAPT-, and VPA-treated mice. (**M** and **N**) Average length (**M**) and average area (**N**) of SG lobes in tail skin from mock-, DAPT-, and VPA-treated mice. (**O**) TEWL in dorsal skin from mock-, DAPT-, and VPA-treated wild-type mice. Unpaired 2-tailed *t* test was used to calculate *P* values in **B**, **D**, **E**, and **G**–**I**. One-way ANOVA with Tukey’s multiple comparisons test was used to calculate *P* values in **M**–**O**.

## Version 1 09/22/2026

In-Press Preview

## Version 2 11/02/2026

Electronic publication
