# Supplementary material for: KROX20 marks sebaceous gland stem cells and regulates their differentiation to maintain skin oil balance
Source: J Clin Invest. 2026 Sep 22;136(21):e206364. doi: 10.1172/JCI206364 (PMC13722400; doi:10.1172/JCI206364)
Supplement: Supplementary file 1 [file jci-136-206364-s126.pdf]

KROX20 marks sebaceous gland stem cells and regulates their differentiation to maintain skin oil balance

Yumeng Zhang[1], Pernelle Pulh[1], Michelle F. Pan[1], Yi He[1], Juanzhu Yan[2], Annie Li[1], Renée M. McKay[1], and Lu Q. Le[1,*]

[1]Department of Dermatology, University of Virginia, Charlottesville, VA 22903, USA.
[2]Department of Cell Biology, University of Texas Southwestern Medical Center, Dallas, TX 75390, USA

[*]Corresponding Author:

Lu Q. Le, M.D., Ph.D.
Professor and Chair
Department of Dermatology
University of Virginia School of Medicine
Phone: (434) 982-5974
Fax: (434) 244-4504
E-mail: bkn6qd@uvahealth.org

Supplemental Figure 1

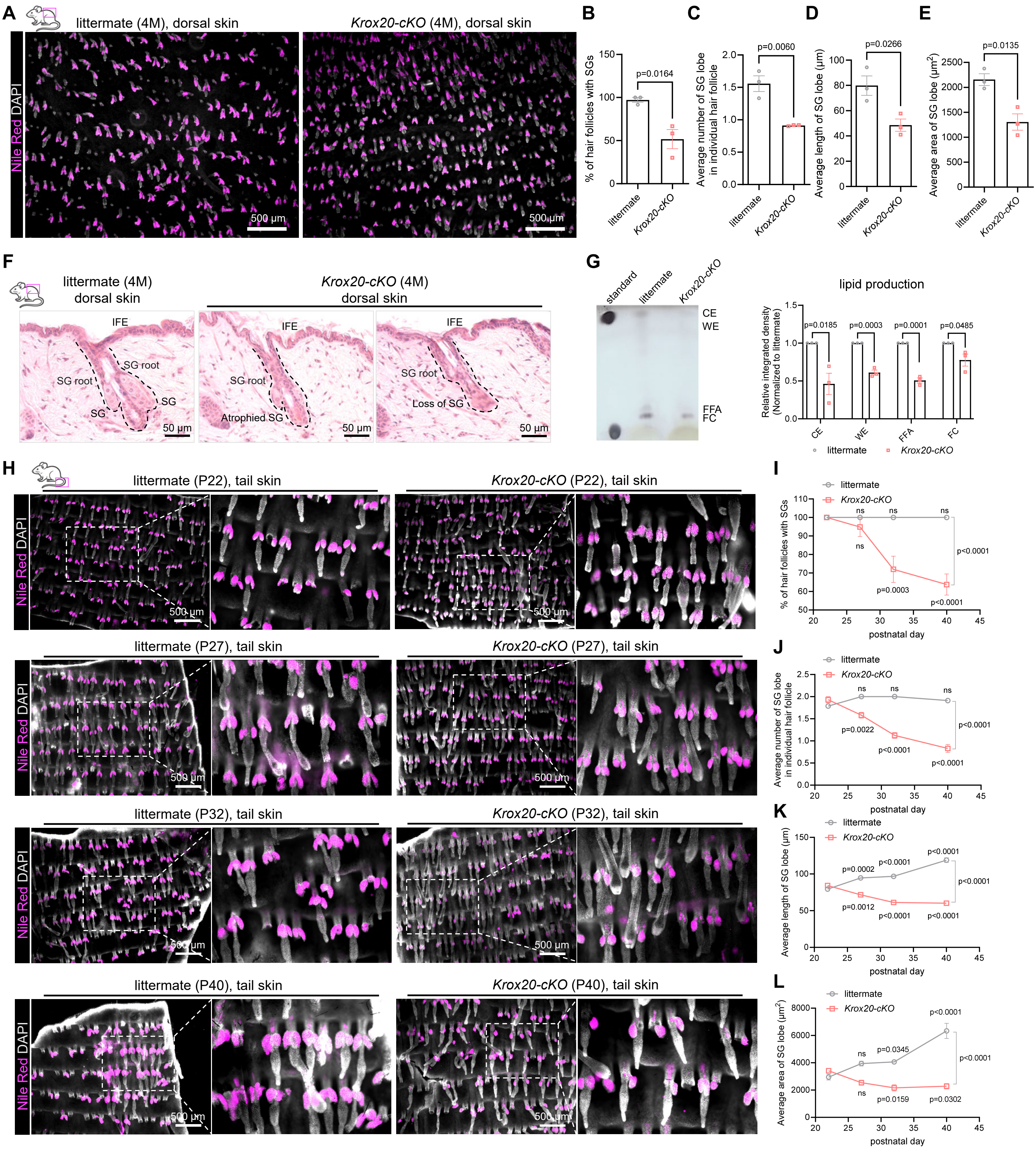

Supplemental Figure 1. *Krox20* dysregulation disrupts SG development and lipid-producing function. (**A**) Representative whole-mount dorsal skin images of SGs from littermate (4M) and *Krox20-cKO* (4M) mice. (**B-E**) Percentage of hair follicles with SGs (**B**), average number of SG lobes in individual hair follicles (**C**), average length (**D**) and average area (**E**) of SG lobes in dorsal skin from littermate (4M) and *Krox20-cKO* (4M) mice. (**F**) H&E staining of dorsal skin paraffin sections from littermate (4M) and *Krox20-cKO* (4M) mice. Atrophied SGs and loss of SGs are shown in *Krox20-cKO* (4M) mice. (**G**) TLC and corresponding quantification of hair lipids (CE, cholesterol esters; WE, wax esters; FFA, free fatty acids; FC, free cholesterol) from littermate (4M) and *Krox20-cKO* (4M) mice. (**H**) Representative whole-mount tail skin images of SGs from littermate and *Krox20-cKO* mice at P22, P27, P32, and P40. (**I-L**) Percentage of hair follicles with SGs (**I**), average number of SG lobes in individual hair follicles (**J**), average length (**K**) and average area (**L**) of SG lobes in tail skin from littermate and *Krox20-cKO* mice at P22, P27, P32, and P40. n = 3 each for littermate and *Krox20-cKO* mice for each time point. Unpaired 2-tailed t test was used to calculate p values in **B-E**. Multiple unpaired t test was used to calculate p values in **G**. Two-way ANOVA with Tukey`s multiple comparisons test was used to calculate p values in **I-L**, with each time point compared to P22, and littermate and *Krox20-cKO* mice compared at P40.

Supplemental Figure 2

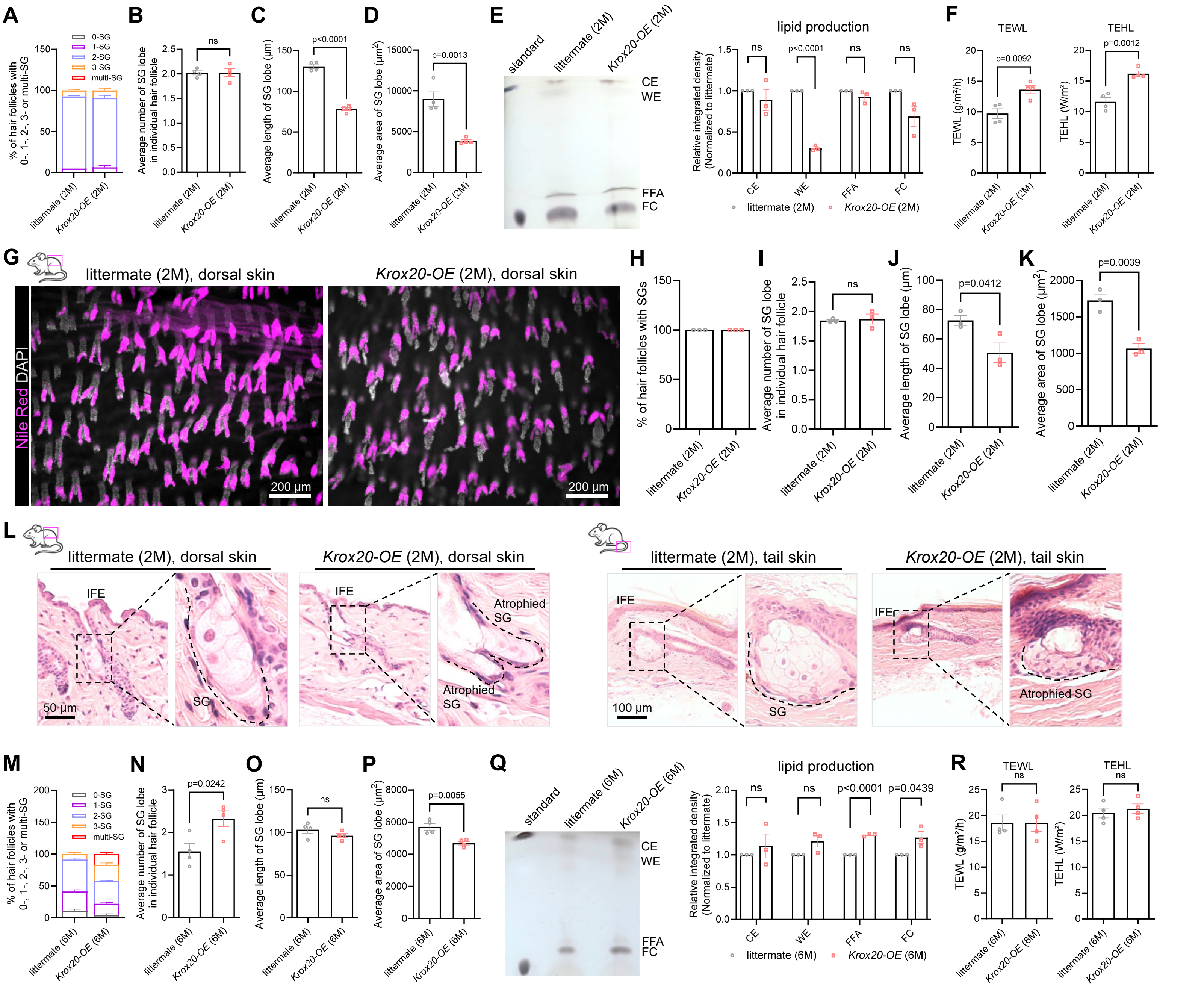

Supplemental Figure 2. Mouse skin displays intrinsic capacity to maintain tissue homeostasis. (**A-D**) Percentage of hair follicles with 0-, 1-, 2-, 3-, or multi-SGs (**A**), average number of SG lobes in individual hair follicles (**B**), average length (**C**) and average area (**D**) of SG lobes in tail skin. (**E**) TLC and corresponding quantification of hair lipids from dorsal skin. (**F**) TEWL and TEHL in dorsal skin. (**G**) Representative whole-mount dorsal skin images of SGs from littermate (2M) and *Krox20-OE* (2M) mice. (**H-K**) Percentage of hair follicles with SGs (**H**), average number of SG lobes in individual hair follicles (**I**), average length (**J**) and average area (**K**) of SG lobes in dorsal skin from littermate (2M) and *Krox20-OE* (2M) mice. (**L**) H&E staining of dorsal and tail skin paraffin sections from littermate (2M) and *Krox20-OE* (2M) mice. (**M-P**) Percentage of hair follicles with 0-, 1-, 2-, 3-, or multi-SGs (**M**), average number of SG lobes in individual hair follicles (**N**), average length (**O**) and average area (**P**) of SG lobes in tail skin. (**Q**) TLC and corresponding quantification of hair lipids from dorsal skin. (**R**) TEWL and TEHL in dorsal skin. Two-way ANOVA with Sidak`s multiple comparisons test was used to calculate p value in **A** and **M**. In **A**, comparisons between 2M littermates and *Krox20-OE* mice were not significant for 0-, 1-, 2-, 3-, or multi-SGs. In **M**, the corresponding p values were: 0-SG, ns; 1-SG, $p=0.0017$; 2-SG, $p=0.0001$; 3-SG, $p<0.0001$; and multi-SG, $p<0.0001$. Multiple unpaired t test was used to calculate p values in **E**, **Q**. Unpaired 2-tailed t test was used to calculate p values in **B-D**, **F**, **H-K**, **N-P**, **R**.

Supplemental Figure 3

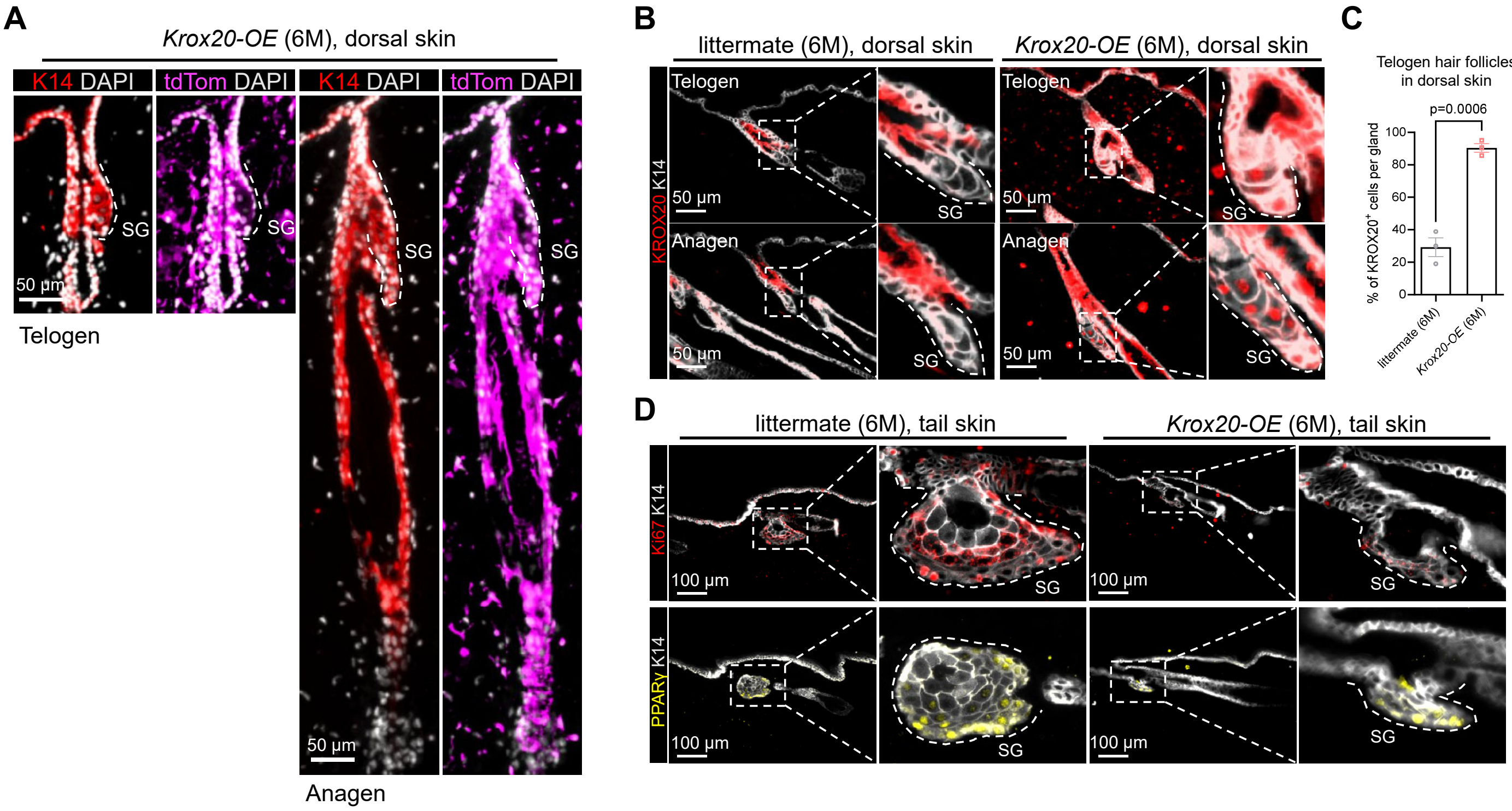

Supplemental Figure 3. *Krox20* overexpression leads to accumulation of SG resident stem cells. (**A**) Paraffin sections of telogen and anagen dorsal skin from littermate (6M) and *Krox20-OE* (6M) mice, co-stained with anti-K14 and anti-tdTomato (tdTom) antibodies. (**B**) Paraffin sections of telogen (upper panel) and anagen (lower panel) dorsal skin from littermate (6M) and *Krox20-OE* (6M) mice, co-stained with anti-K14 and anti-KROX20 antibodies. (**C**) Percentage of $KROX20^+$ cells in telogen (left panel) and anagen (right panel) dorsal skin SGs of littermate (6M) and *Krox20-OE* (6M) mice. (**D**) Paraffin sections of tail skin from littermate (6M) and *Krox20-OE* (6M) mice, co-stained with anti-K14/ anti-Ki67 antibodies or anti-K14/ anti-PPARγ antibodies. Unpaired 2-tailed t test was used to calculate p value in **C**. Hair cycle was matched for quantifications in **D**.

Supplemental Figure 4

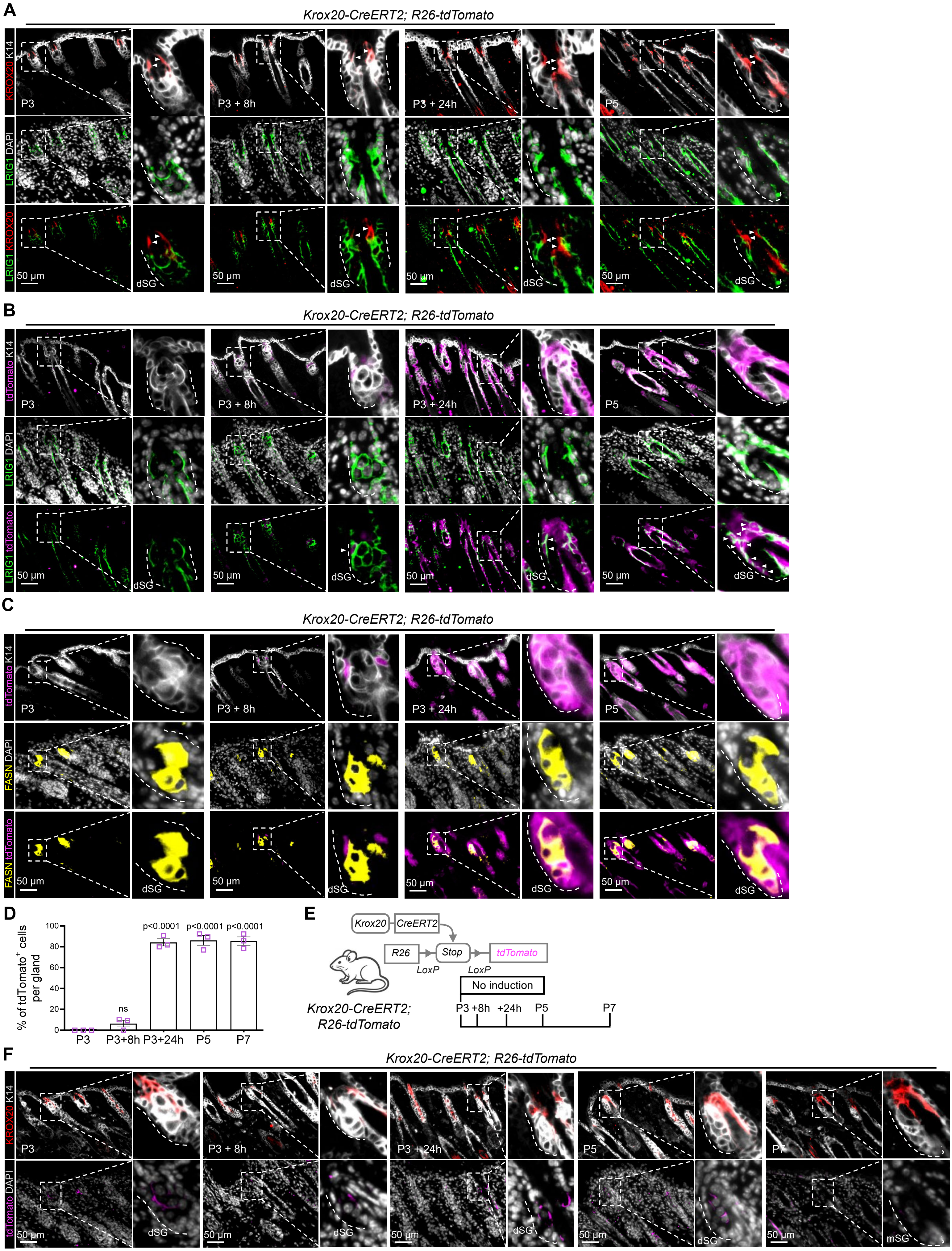

Supplemental Figure 4. *Krox20*-lineage cells govern cellular input to the developing SGs. (**A-C**) Paraffin sections of dorsal skin from *Krox20-CreERT2; R26-tdTomato* mice at P3, P3 + 8h, P3 + 24h, and P5. Mice were co-stained with anti-K14, anti-KROX20, and anti-LRIG1 antibodies. $KROX20^+$ cells indicated with white arrowheads (**A**); co-stained with anti-K14, anti-LRIG1, and anti-tdTomato antibodies. $tdTomato^+$/ $LRIG1^+$ cells indicated with white arrowheads (**B**); co-stained with anti-K14, anti-FASN, and anti-tdTomato antibodies (**C**). (**D**) Percentage of *Krox20*-lineage cells ($tdTomato^+$ cells) in SGs during SG morphogenesis. n = 3 for each time point. (**E**) Scheme for *Krox20*-lineage tracing during SG morphogenesis in inducible *Krox20-CreERT2; R26-tdTomato* mice without induction at P3. (**F**) Paraffin sections of dorsal skin from *Krox20-CreERT2; R26-tdTomato* mice at P3, P3 + 8h, P3 + 24h, P5, and P7. Mice were co-stained with anti-K14, anti-KROX20, and anti-tdTomato antibodies. One-way ANOVA with Tukey`s multiple comparisons test was used to calculate p values in **D**, with each time point compared to P3.

Supplemental Figure 5

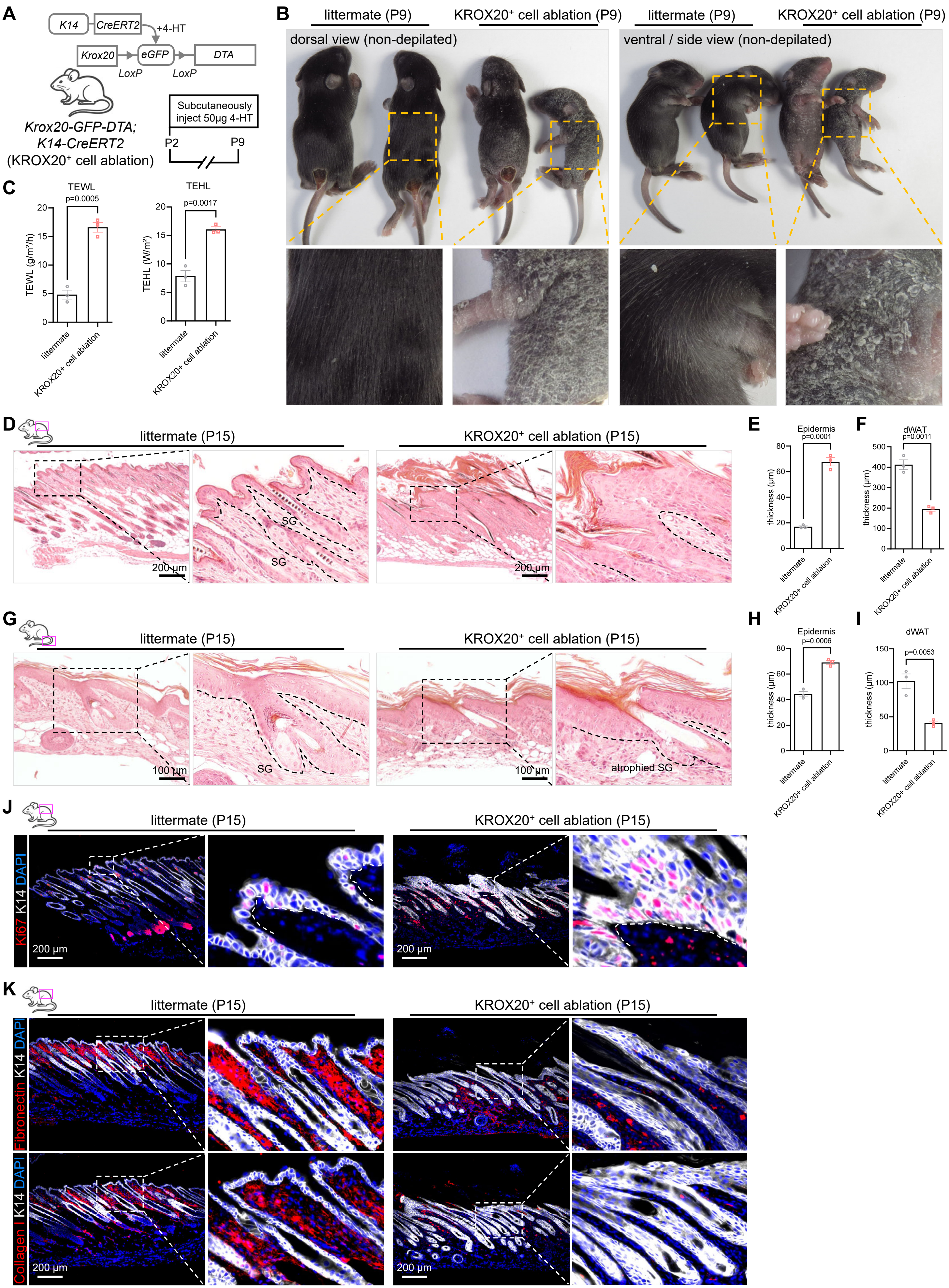

Supplemental Figure 5. Ablation of KROX20$^+$ cells leads to dry, flaky, alopecic skin within 7 days. (A) Scheme for ablating KROX20$^+$ cells during SG morphogenesis in *Krox20-GFP-DTA; K14-CreERT2* (KROX20$^+$ cell ablation) mice. Mice were induced at P2 and harvested at P9. n = 3 for each genotype. (**B**) Dorsal view (non-depilated) and ventral/ side view (non-depilated) from littermate (P9) and KROX20$^+$ cell ablation (P9) mice. (**C**) TEWL and TEHL in dorsal skin from littermate (P9) and KROX20$^+$ cell ablation (P9) mice. (**D**) H&E staining of dorsal skin paraffin sections from littermate (P15) and KROX20$^+$ cell ablation (P15) mice. (**E-F**) Quantification of epidermal thickness (**E**) and dermal white adipose tissue (dWAT) thickness (**F**) based on the histological data in (**D**). (**G**) H&E staining of tail skin paraffin sections from littermate (P15) and KROX20$^+$ cell ablation (P15) mice. (**H-I**) Quantification of epidermal thickness (**H**) and dWAT thickness (**I**) based on the histological data in (**G**). (**J**) Paraffin sections of dorsal skin from littermate (P15) and KROX20$^+$ cell ablation (P15) mice, co-stained with anti-K14 and anti-Ki67 antibodies. (**K**) Paraffin sections of dorsal skin from littermate (P15) and KROX20$^+$ cell ablation (P15) mice, co-stained with anti-K14/ anti-Fibronectin and anti-K14/ anti-Collagen I antibodies. Unpaired 2-tailed t test was used to calculate p values in **C**, **E-F**, **H-I**.

Supplemental Figure 6

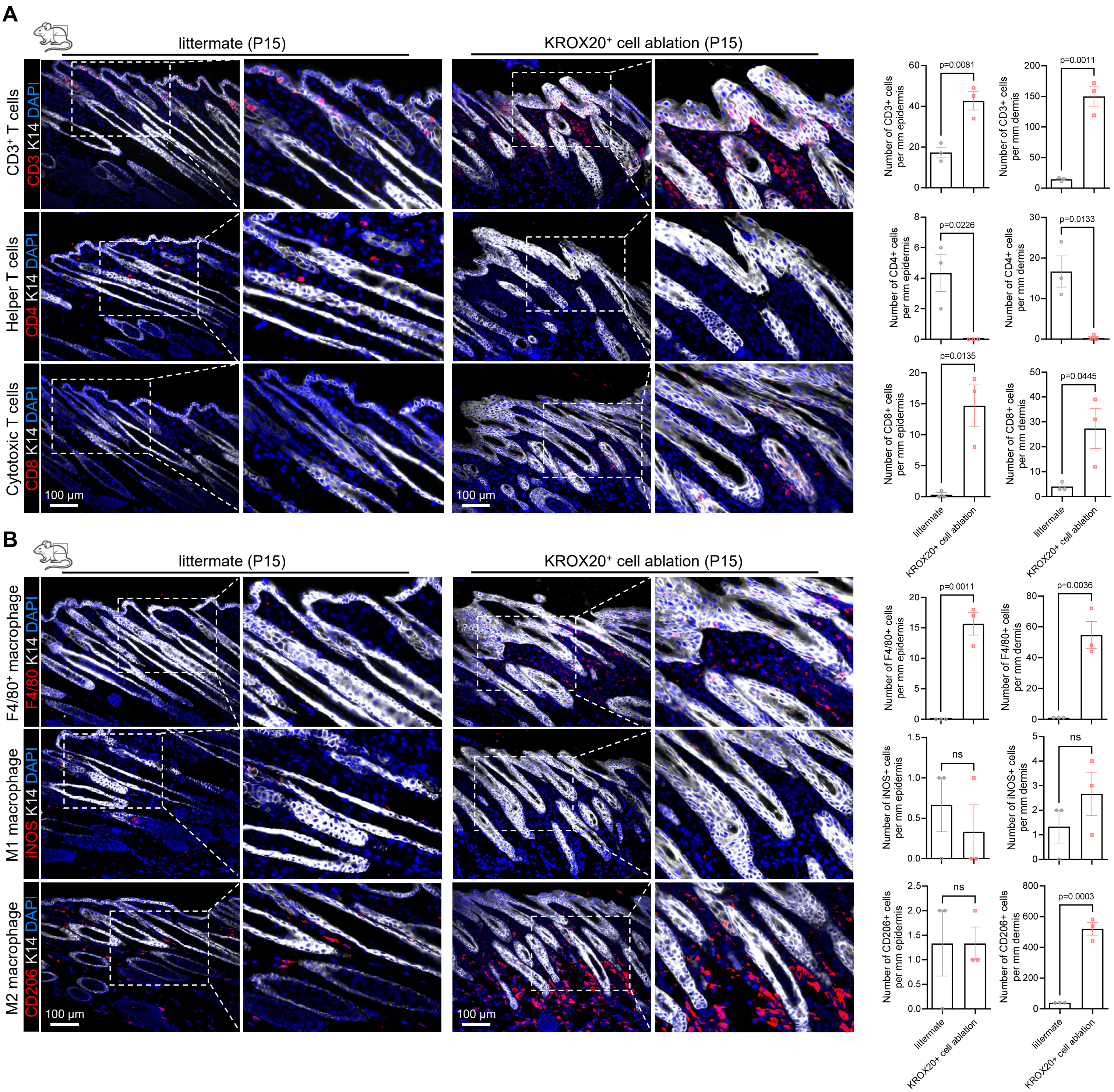

Supplemental Figure 6. Loss of SGs caused by KROX20$^+$ cell ablation leads to immune infiltration. (**A**) T cell profiling: paraffin sections of dorsal skin from littermate (P15) and KROX20$^+$ cell ablation (P15) mice, co-stained with anti-K14/ anti-CD3 (pan T cell marker), anti-K14/ anti-CD4 (helper T cell marker), and anti-K14/ anti-CD8 (cytotoxic T cell marker) antibodies (left panel), and quantification of CD3$^+$, CD4$^+$, and CD8$^+$ cells in epidermis and dermis (right panel). (**B**) Macrophage cell profiling: paraffin sections of dorsal skin from littermate (P15) and KROX20$^+$ cell ablation (P15) mice, co-stained with anti-K14/ anti-F4/80 (mouse macrophage marker), anti-K14/ anti-iNOS (M1 macrophage), and anti-K14/ anti-CD206 (M2 macrophage) antibodies (left panel), and quantification of F4/80$^+$, iNOS$^+$, CD206$^+$ cells in epidermis and dermis (right panel). Unpaired 2-tailed t test was used to calculate p values in **A-B**.

Supplemental Figure 7

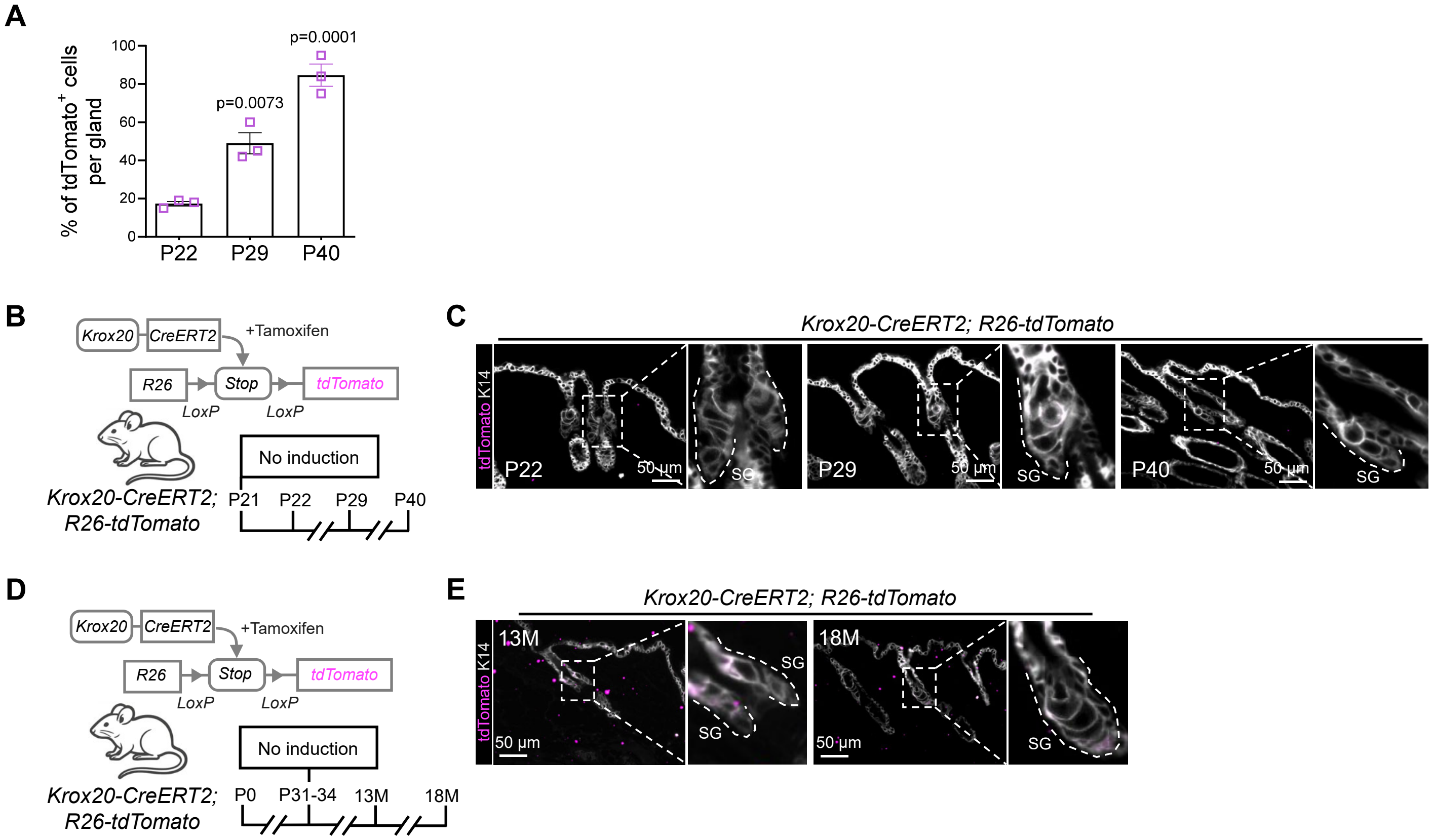

Supplemental Figure 7. Cre recombinase is tightly regulated in inducible *Krox20-CreERT2; R26-tdTomato* mice. (**A**) Percentage of *Krox20*-lineage cells (tdTomato^{+} cells) in SGs during SG homeostasis. n = 3 for each time point. (**B**) Scheme for *Krox20*-lineage tracing during SG homeostasis in inducible *Krox20-CreERT2; R26-tdTomato* mice without tamoxifen induction at P21. Mice were harvested at P22, P29 and P40. (**C**) Paraffin sections of dorsal skin from *Krox20-CreERT2; R26-tdTomato* mice at P22, P29, and P40 co-stained with anti-K14 and anti-tdTomato antibodies. (**D**) Scheme for *Krox20*-lineage tracing during SG homeostasis in inducible *Krox20-CreERT2; R26-tdTomato* mice without tamoxifen induction at P31-P34. Mice were harvested at 13M and 18M. (**E**) Paraffin sections of dorsal skin from *Krox20-CreERT2; R26-tdTomato* mice at 13M and 18M, co-stained with anti-K14 and anti-tdTomato antibodies. One-way ANOVA with Tukey`s multiple comparisons test was used to calculate p values in **A**, with each time point compared to P22.

Supplemental Figure 8

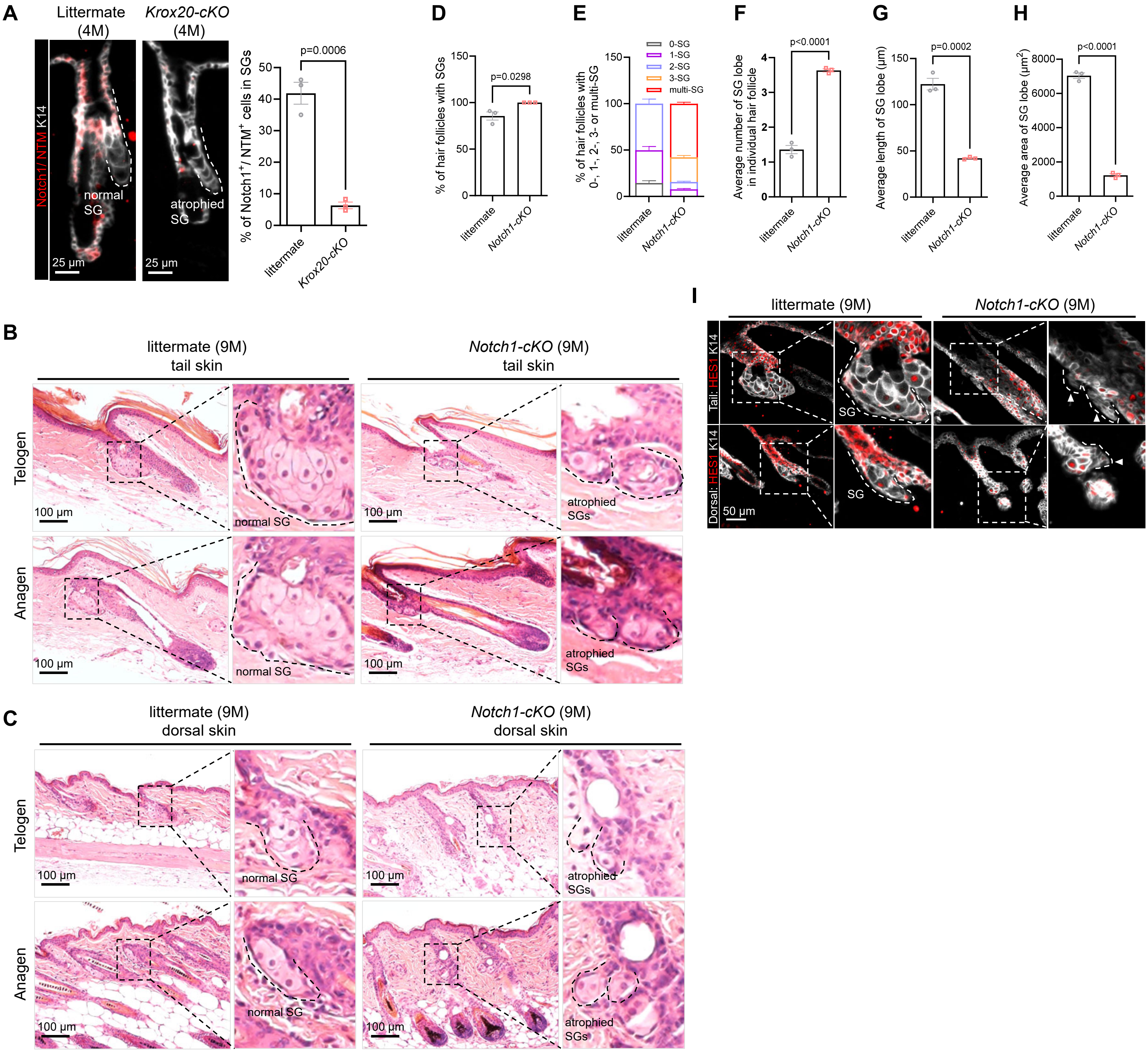

Supplemental Figure 8. Loss of *Notch1* in *Krox20*-expressing cells results in multi-lobed, atrophied SGs in both telogen and anagen skin. (**A**) Paraffin sections of dorsal skin from littermate (4M) and *Krox20-cKO* (4M) mice, co-stained with anti-K14 and anti-Notch1/ NTM antibodies (left panel). Corresponding percentage of Notch1^{+}/ NTM^{+} cells in SGs (right panel). (**B**) H&E staining of telogen (upper panels) and anagen (lower panels) tail skin paraffin sections from littermate (9M) and *Notch1-cKO* (9M) mice. (**C**) H&E staining of telogen (upper panels) and anagen (lower panels) dorsal skin paraffin sections from littermate (9M) and *Notch1-cKO* (9M) mice. Black dashed lines demarcate normal SGs in littermate (9M) mice and multi-lobed atrophied SGs in *Notch1-cKO* (9M) mice. (**D-H**) Percentage of hair follicles with SGs (**D**), percentage of hair follicles with 0-, 1-, 2-, 3-, or multi-SGs (**E**), average number of SG lobes in individual hair follicles (**F**), average length (**G**) and average area (**H**) of SG lobes in tail skin from littermate (9M) and *Notch1-cKO* (9M) mice. (**I**) Paraffin sections of dorsal and tail skin from littermate (9M) and *Notch1-cKO* (9M) mice, co-stained with anti-K14/ anti-HES1 antibodies. Ectopically developed atrophied SG lobes denoted with white arrow heads. Unpaired 2-tailed t test was used to calculate p values in **A**, **D**, **F-H**. Two-way ANOVA with Sidak`s multiple comparisons test was used to calculate p value in **E**, the corresponding p values were: 0-SG, $p=0.0014$; 1-SG, $p<0.0001$; 2-SG, $p<0.0001$; 3-SG, $p<0.0001$; and multi-SG, $p<0.0001$. Hair cycle was matched for quantifications in **A** and **I**.

Supplemental Figure 9

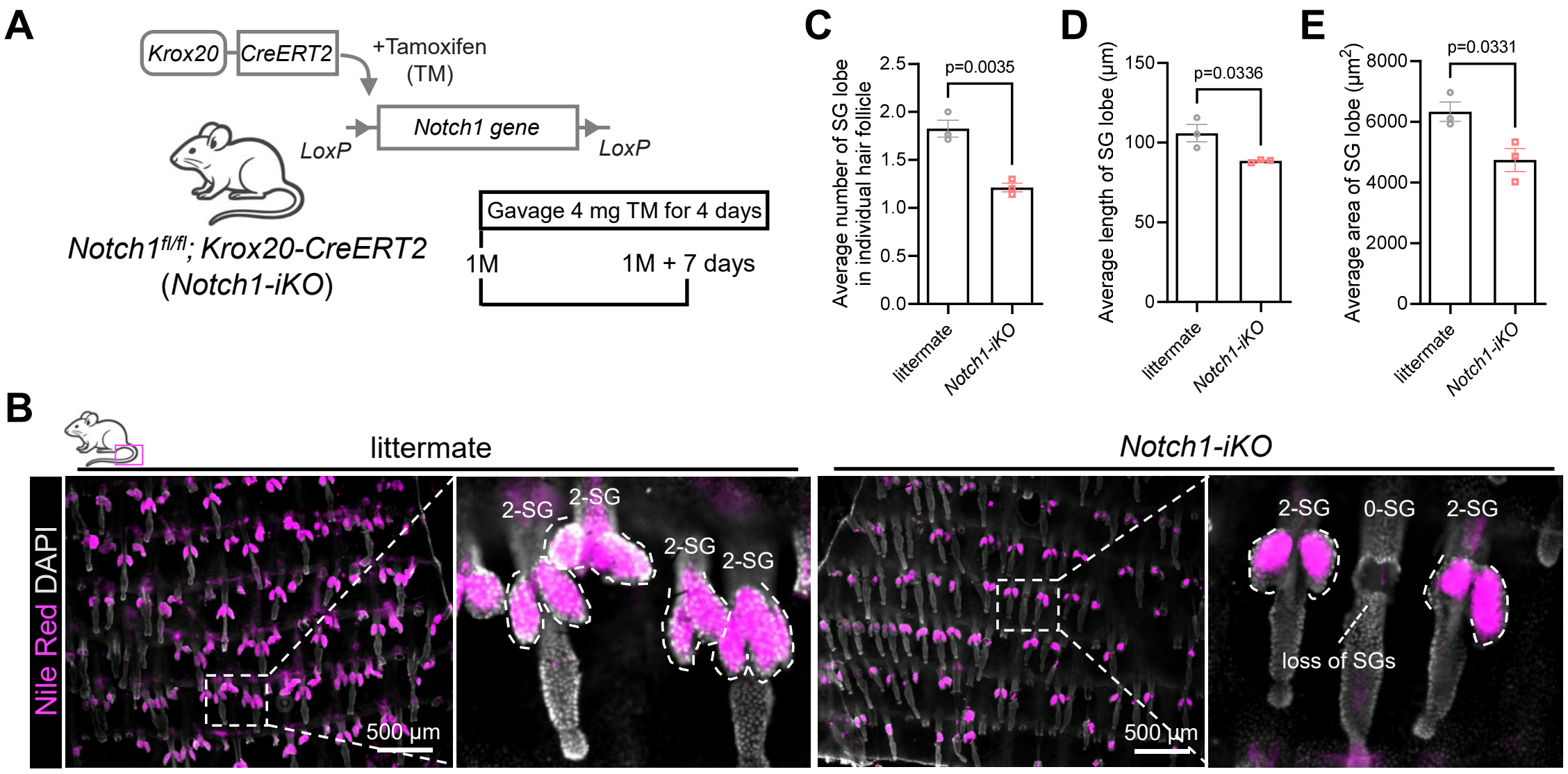

Supplemental Figure 9. Inducible *Notch1* knockout in *Krox20*-expressing cells results in rapid SG morphology changes. (**A**) Scheme for deleting *Notch1* in *Krox20*-expressing cells in *Notch1$^{fl/fl}$; Krox20-CreERT2* (*Notch1-iKO*) mice. Mice were induced at 1M with 4 mg tamoxifen for 4 days and analyzed 7 days later (1M + 7 days). n = 3 for each genotype. (**B**) Representative whole-mount tail skin images of SGs from littermate (1M + 7 days) and *Notch1-iKO* (1M + 7 days) mice. (**C-E**) Average number of SG lobes in individual hair follicles (**C**), average length (**D**) and average area (**E**) of SG lobes in tail skin from littermate (1M + 7 days) and *Notch1-iKO* (1M + 7 days) mice. Unpaired 2-tailed t test was used to calculate p values in **C-E**.

Supplemental Figure 10

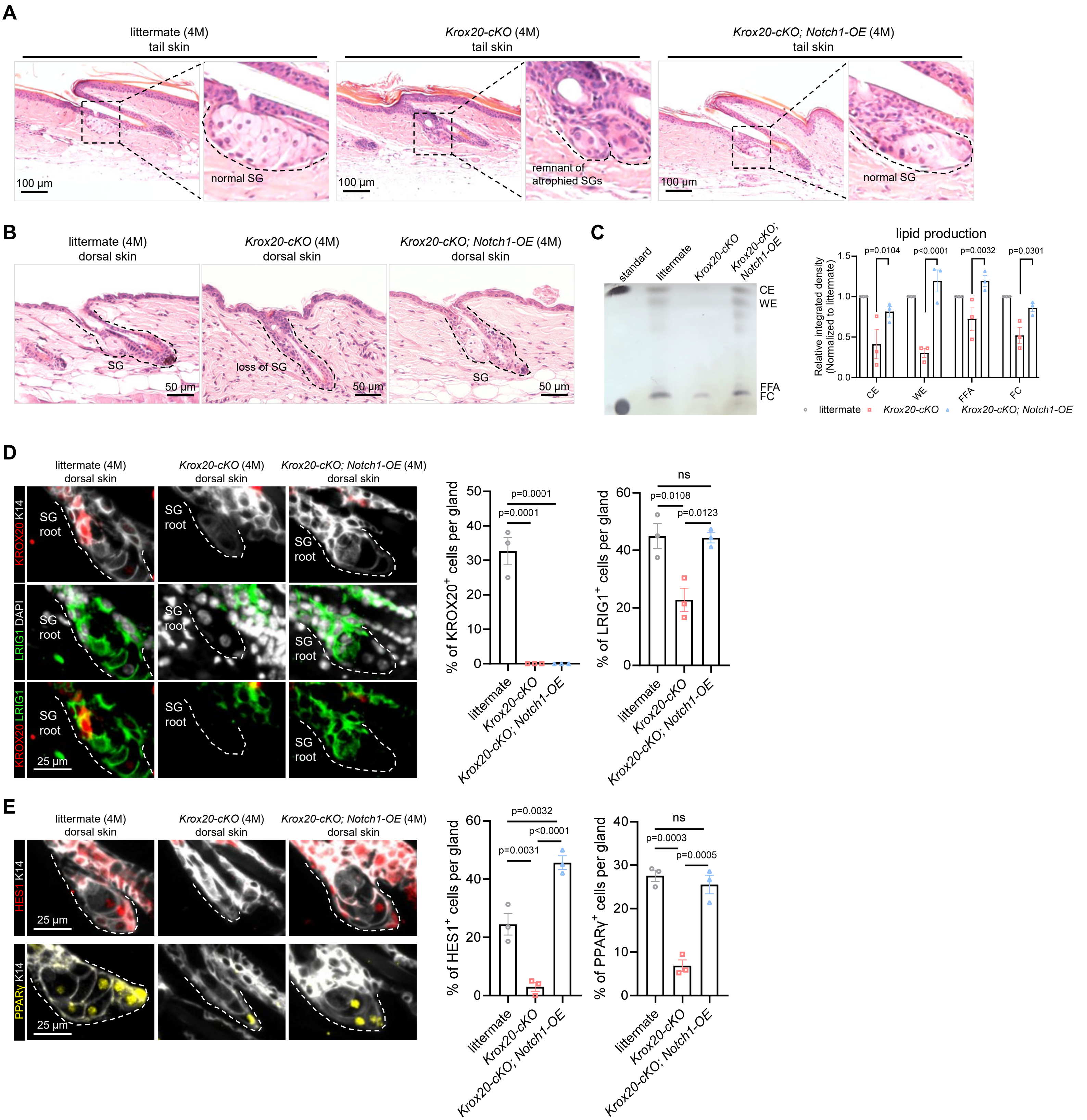

Supplemental Figure 10. *Notch1* overexpression restores SG lipid-producing function. (**A-B**) H&E staining of tail skin (**A**) and dorsal skin (**B**) paraffin sections from littermate (4M), *Krox20-cKO* (4M), and *Krox20-cKO; Notch1-OE* (4M) mice. (**C**) Thin Layer Chromatography and corresponding quantification of hair lipids (CE, cholesterol esters; WE, wax esters; FFA, free fatty acids; FC, free cholesterol) from littermate (4M), *Krox20-cKO* (4M), and *Krox20-cKO; Notch1-OE* (4M) mice. (**D**) Paraffin sections of dorsal skin from littermate (4M), *Krox20-cKO* (4M), and *Krox20-cKO; Notch1-OE* (4M) mice, co-stained with anti-K14, anti-KROX20, and anti-LRIG1 antibodies (left panel), and quantification of percentage of KROX20^{+} and LRIG1^{+} cells per gland (right panel). (**E**) Paraffin sections of dorsal skin from littermate (4M), *Krox20-cKO* (4M), and *Krox20-cKO; Notch1-OE* (4M) mice, co-stained with anti-K14/ anti-HES1 and anti-K14/ anti-PPARγ antibodies (left panel). Quantification of percentage of HES1^{+} and PPARγ^{+} cells per gland (right panel). Two-way ANOVA with Tukey`s multiple comparisons test was used to calculate p values in **C**. One-way ANOVA with Tukey`s multiple comparisons test was used to calculate p values in **D-E**. Hair cycle was matched for quantifications in **D** and **E**.

Supplemental Figure 11

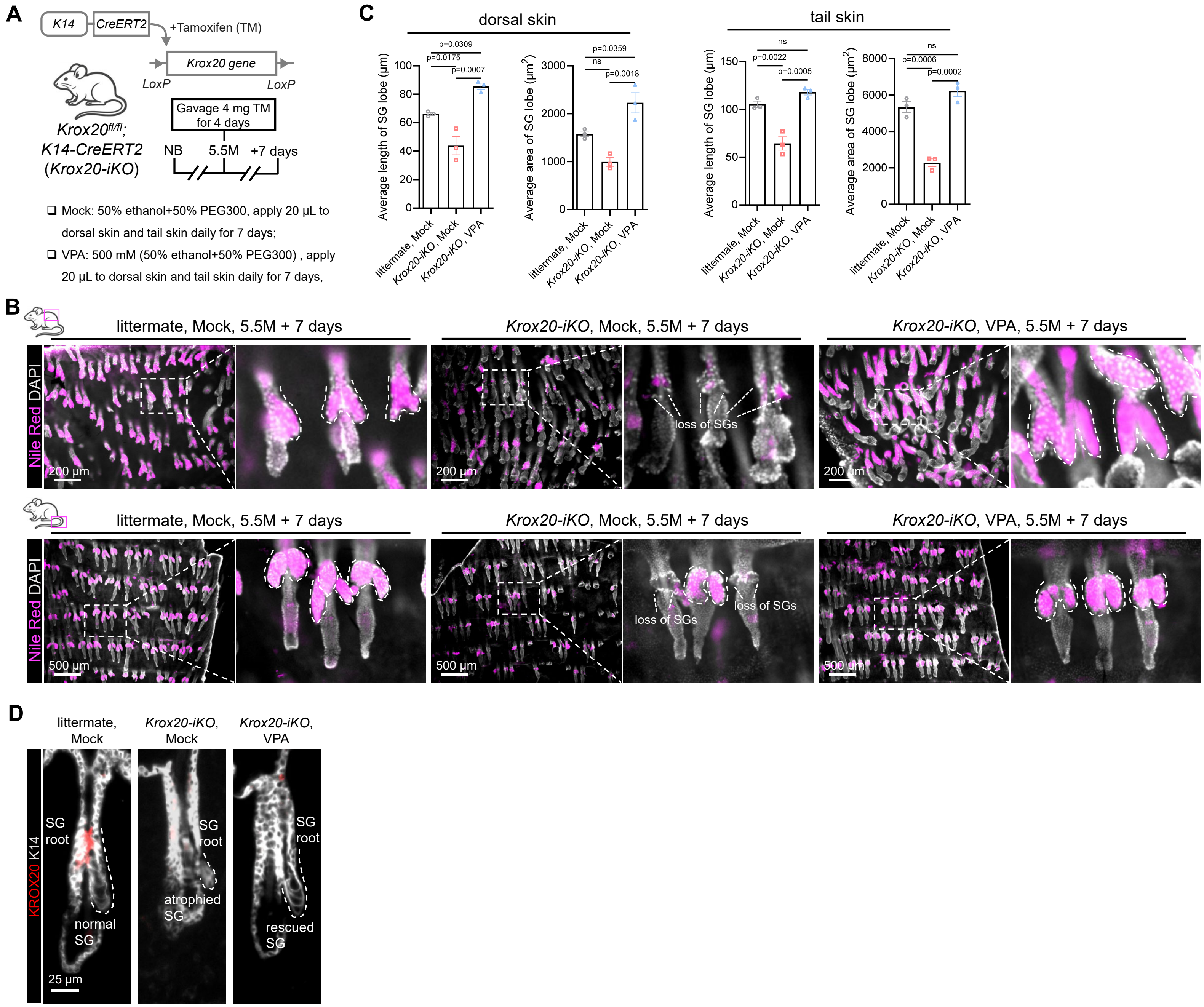

Supplemental Figure 11. Pharmacological activation of *Notch1* signaling rescues the atrophied SG lobes resulting from loss of *Krox20*. (**A**) Schematic illustrating experimental design for pharmacological activation of the *Notch1* signaling in inducible *Krox20$^{fl/fl}$; K14-CreERT2* (*Krox20-iKO*) mice. Mice were induced at 5.5M, and vehicle (Mock) or VPA were topically applied daily to dorsal skin. Mice were euthanized 7 days later (5.5M + 7 days). (**B**) Representative images of whole-mount dorsal (upper panel) and tail (lower panel) skin SGs from littermate (Mock, 5.5M + 7 days), *Krox20-iKO* (Mock, 5.5M + 7 days), *Krox20-iKO* (VPA, 5.5M + 7 days) mice. n = 3 for each genotype. (**C**) Average length and average area of SG lobes in dorsal skin (left panel) and in tail skin (right panel) based on whole-mount imaging in (**B**). (**D**) Paraffin sections of dorsal skin from littermate (mock, 5.5M + 7 days), *Krox20-iKO* (mock, 5.5M + 7 days), *Krox20-iKO* (VPA, 5.5M + 7 days) mice, co-stained with anti-K14/ anti-KROX20. One-way ANOVA with Tukey`s multiple comparisons test was used to calculate p values in **C**. Hair cycle was matched for quantifications in **D**.

Supplemental Figure 12

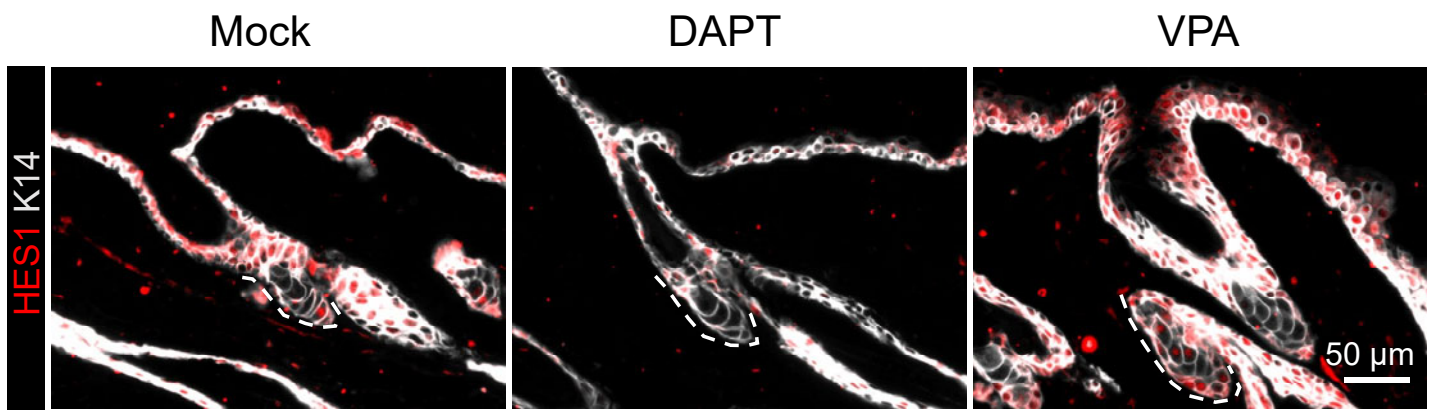

Supplemental Figure 12. *Notch1* signaling inhibitor and activator respectively downregulates and upregulates *Notch1* signaling. (**A**) Paraffin sections of dorsal skin from mock-, DAPT-, and VPA-treated wild type mice, co-stained with anti-K14/ anti-HES1 antibodies. Hair cycle was matched for quantifications in **A**.

Supplemental Figure 13

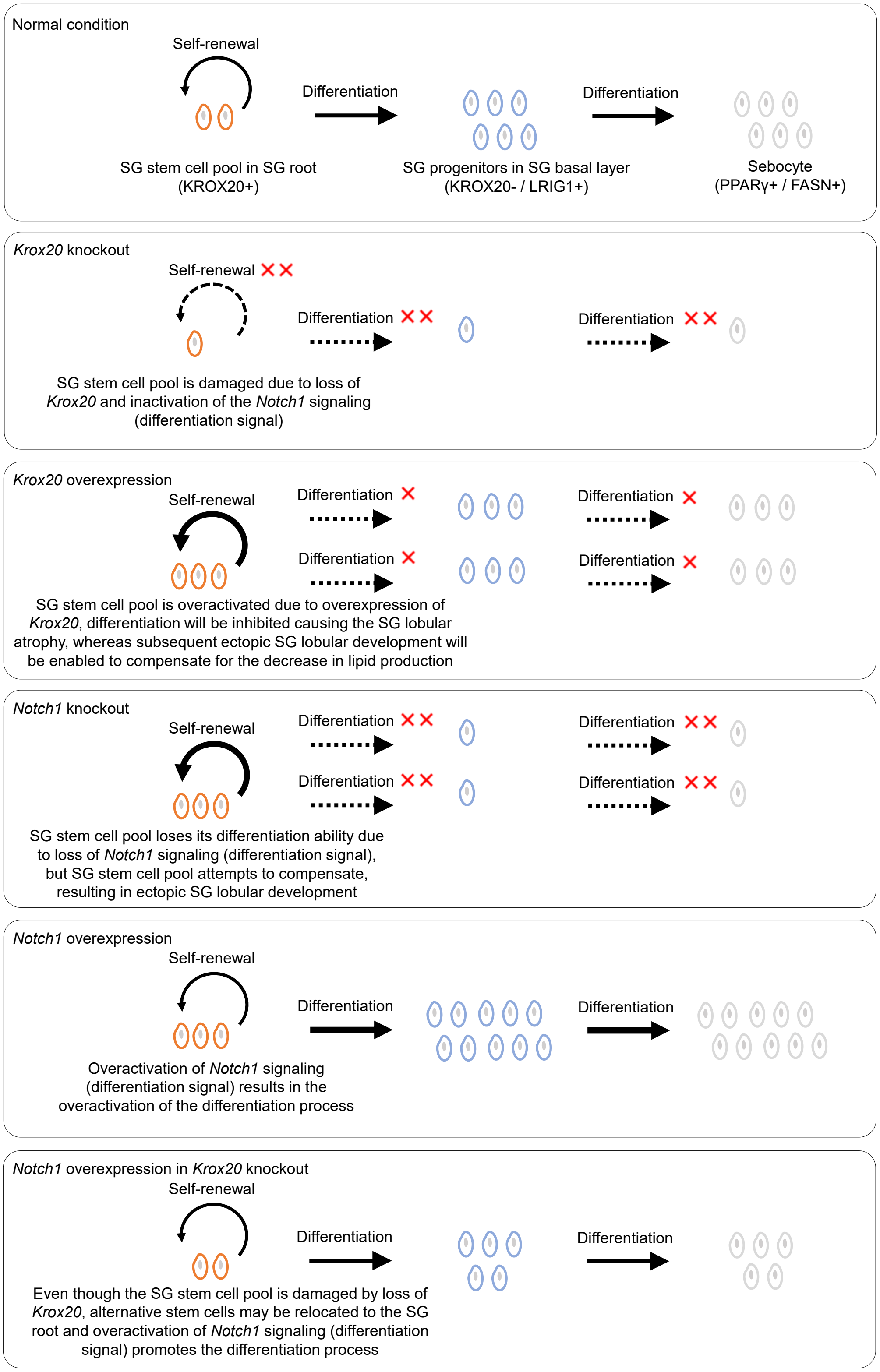

Supplemental Figure 13. Summary for hierarchical process from SG stem cells to sebocytes under control of *Krox20* and *Notch1* signaling. Diagram illustrating the hierarchical process from SG stem cells to sebocytes under normal conditions, *Krox20* knockout, *Krox20* overexpression, *Notch1* knockout, *Notch1* overexpression, and *Notch1* overexpression in *Krox20* knockout scenarios to dissect the function of *Krox20* as a self-renewal signal and *Notch1* as a differentiation signal.
